# Clinical Significance and Immune Landscape of Recurrence-Associated Ferroptosis Signature in Early-Stage Lung Adenocarcinoma

**DOI:** 10.3389/fonc.2022.794293

**Published:** 2022-01-27

**Authors:** Lilan Yi, Ping Huang, Yinfang Gu, Guowu Wu, Xiaofang Zou, Longhua Guo, Chunling Wen, Junlin Zhu, Dongdong Zhao

**Affiliations:** ^1^ Department of Oncology, Cancer Center, Meizhou People’s Hospital (Huangtang Hospital), Meizhou Academy of Medical Sciences, Meizhou, China; ^2^ Guangdong Provincial Key Laboratory of Precision Medicine and Clinical Translational Research of Hakka Population, Meizhou, China

**Keywords:** early-stage lung adenocarcinoma, recurrence, ferroptosis signature, immune landscape, immunotherapy

## Abstract

**Background:**

The prevalence of patients newly diagnosed with early-stage lung adenocarcinoma (LUAD) is growing alongside significant advances in screening approaches. This study aimed to construct ferroptosis-related gene score (FRGscore) for predicting recurrence, explore immune-molecular characteristics, and determine the benefit of immunotherapy in distinct ferroptosis-based patterns and FRGscore-defined subgroups.

**Methods:**

A total of 1,085 early-stage LUAD patients from four independent cohorts were included. Consensus clustering analysis was performed using 217 co-expressed FRGs to explore different ferroptosis-mediated patterns. An FRG scoring system was established to predict relapse, quantify ferroptosis-mediated patterns, and evaluate the response to immunotherapy in individual patients based on Lasso-penalized and stepwise Cox regression analyses. Immune landscape involving multiple parameters was further evaluated, stratified by cluster subtypes and FRGscore subgroups.

**Results:**

Two ferroptosis-mediated patterns were identified and verified, which were characterized by significantly distinct prognosis and immune profiles. Analyses of immune characteristics showed that identified ferroptosis patterns were characterized as immune-inflamed phenotype and immune-exhausted phenotype. The FRG scoring model based on 11 FRG-derived signatures panel classified patients into the FRGscore-high and FRGscore-low subgroups. Significantly longer recurrence-free survival (RFS) and overall survival (OS) were observed in the FRGscore-low subgroup. FRGscore-low patients were characterized by higher tumor mutational burden (TMB), immunoscore, immunophenoscore, and PD-L1 expression level and were associated with lower Tumor Immune Dysfunction and Exclusion (TIDE) score, whereas the opposite was observed in FRGscore-high patients. Immune-active pathways were remarkably enriched in the FRGscore-low subgroup. This scoring model remained highly predictive of prognosis across different clinical, molecular, and immune subgroups. Further analysis indicated that FRGscore-low patients exhibited higher response to anti-PD-1/PD-L1 immunotherapy and better clinical benefits based on two independent immunotherapy cohorts.

**Conclusion:**

The proposed FRGscore could highly distinguish the recurrence patterns and molecular and immune characteristics and could predict immunotherapy prognosis, potentially representing a powerful prognostic tool for further optimization of individuated treatment and management strategies in early-stage LUAD.

## Introduction

Lung cancer is the leading cause of cancer-associated deaths worldwide and is characterized by high incidence and mortality (1). The cancer statistics released in 2020 showed that the number of new lung cancer cases and deaths worldwide was approximately 2.2 million and 1.8 million, respectively (1). Lung adenocarcinoma (LUAD) is the predominant pathological subtype associated with the high incidence and death cases attributed to lung cancer, representing more than 40% of all lung cancer cases (2). The survival rate in early-stage LUAD remains bleak owing to the high incidence of recurrence and metastasis after surgery, with a 5-year recurrence rate of approximately 30%–45% (3). The prevalence of early-stage cases has increased due to advances in lung cancer screening strategies (4, 5). Therefore, it is crucial to develop optimal strategies for treatment and management of early-stage diseases, especially to screen those patients at high risk of recurrence who urgently need additional personalized adjuvant therapy.

Ferroptosis is an iron-dependent novel type of programmed cell death different from apoptosis, necrosis, and autophagy (6, 7). The main mechanism of ferroptosis is through the action of divalent iron or ester oxygenase that catalyzes unsaturated fatty acids overexpressed on the cell membrane, which are highly vulnerable to lipid peroxidation, thus inducing cell death. A study by Lu et al. reported that ferroptosis was intricately involved in cancer development, tumor suppression process, and therapeutic response (8). Ferroptosis inhibits tumor growth by regulating metabolism and promoting cell death (9). Lai et al. displayed that the ferroptosis process of lung tissue was inhibited by upregulation of reverse transporter protein and decrease in the level of iron ions, resulting in occurrence and development of lung cancer (10). Previous studies revealed that modulation of ferroptosis progression affected proliferation, colony formation, and cell death of lung cancer cells and was associated with the prognosis of LUAD patients (11, 12). However, the clinical outcomes, genomic features, and treatment responses of different ferroptosis-based patterns have not yet been explored in patients with early-stage LUAD. In addition, robust biomarkers are warranted to be identified to guide development of personalized adjuvant therapies for early-stage LUAD patients.

Immune checkpoint inhibitor (ICI) therapies have been used in cancer treatment to block T-cell inhibitory molecules, showing positive results, and have the potential to improve the prognosis of cancer; however, not all patients benefit from ICI therapies. Therefore, several studies are currently underway to improve the efficacy of cancer immunotherapy. Previous *in vivo* and *in vitro* studies uncovered that dysregulation of ferroptosis may play a crucial role in cancer drug resistance and tumor immune escape (13). Cancer cells present with an increased demand for iron over normal non-cancer cells; therefore, induction of ferroptosis is a promising treatment method to effectively trigger cancer cells death, mainly in tumors that are resistant to traditional therapies (14). Wang et al. reported that CD8+ T cells activated by immunotherapy induce ferroptosis of tumor cells to enhance the antitumor activity of immunotherapy (15). Moreover, ferroptosis is immunogenic and could potentially induce inflammatory immune response, which is characterized by immunogenic cell death (16). These findings indicate that ferroptosis is correlated with antitumor immunity. The study of Gao et al. and Zhang et al. indicated that ferroptosis-related signatures have the role of predicting prognosis in LUAD patients. However, the immunotherapy prognosis role was not analyzed in LUAD. In addition, whether FRG-driven signatures are correlated with PD-L1 remains largely elusive (17, 18). Currently, the immune characteristics and response to immunotherapy of different ferroptosis-mediated patterns in early-stage LUAD have not been elucidated.

Therefore, it is imperative to establish recurrence-associated signatures based on ferroptosis-related gene (FRG) in early-stage LUAD owing to the key role of ferroptosis in antitumor immune regulation and an increasing number of early-stage LUAD patients at first diagnosis. In the current study, recurrence-free survival (RFS) data of 1,085 early-stage LUAD patients from four independent cohorts were integrated to develop and validate FRG-based recurrence signatures. Furthermore, clinicopathological features and immune characteristics of FRG-driven signatures were explored. The proposed FRG-based scoring system for early-stage LUAD provides an understanding of the complex underlying mechanism between ferroptosis and LUAD recurrence and provides a basis for the development of tailoring immunotherapy strategies for early-stage diseases.

## Materials and Methods

### Dataset Acquisition and Preprocessing

RNA-seq expression data, somatic mutation data, and corresponding clinical parameters of LUAD patients were retrieved from The Cancer Genome Atlas (TCGA; https://portal.gdc.cancer.gov) database. Data for a total of 369 stage I–II LUAD patients including overall survival (OS) and RFS data were retrieved and were used as the training cohort. Three datasets—GSE31210 (n = 226), GSE68465 (n = 363), and GSE50081 (n = 127)—were extracted from Gene Expression Omnibus (GEO; https://www.ncbi.nlm.nih.gov/geo/) using the same inclusion criteria for use as independent validation cohorts. The mRNA expression data for the three validation cohorts were log2 transformed and quantile normalized; then the average expression level of genes determined by multiple probes was calculated. Details on baseline characteristics of the included four publicly available datasets are presented in **Tables S1** and **S2**. Clinical parameters including age, gender, smoking history, TNM stage, epidermal growth factor receptor (EGFR) status, KRAS status, RFS, and OS were retrieved. A total of 274 FRGs were obtained from the published literature and the ferroptosis database (http://www.zhounan.org/ferrdb) (6, 14, 19–21). The selected FRGs and mRNA expression profiles of eligible LUAD datasets were matched and overlapped, resulting in 217 co-expressed FRGs.

### Consensus Clustering for Ferroptosis-Related Genes

Hierarchical agglomerative clustering was conducted using “ConsensusClusterPlus” (http://www.bioconductor.org/) to explore the functional patterns of FRGs in early-stage LUAD. The unsupervised clustering with Euclidean and Ward’s linkage was employed to classify early-stage LUAD samples in TCGA cohort into different clustering subtypes, and 1,000 replicates were performed to ensure that the classifications were reliable. The cluster subtypes were verified using the same method for the GSE31210 cohort. Kyoto Encyclopedia of Genes and Genomes (KEGG) pathway analysis was carried out using the clusterProfiler R package to further explore the biological functions of distinct ferroptosis patterns mediated by FRGs.

### Generation and Validation of Ferroptosis-Related Gene Score

Further analysis was performed to comprehensively evaluate the association between ferroptosis and tumor recurrence in early-stage LUAD and develop robust discrimination criteria based on FRG-driven signatures (**Figure S1**). First, a univariate Cox regression model was utilized to explore the correlation between each FRG expression and RFS. Subsequently, Lasso-penalized Cox regression analysis was conducted for variable selection to determine significant prognostic genes (22). Stepwise Cox proportional hazards regression model was further implemented to optimize the established model. Finally, a scoring system based on 11 FRG relevant signatures was constructed using a linear combination of regression coefficients yielded by Cox regression analysis and the optimized FRG expression level, which was termed as FRGscore. The established FRGscore formula was as follows: FRGscore = sum of coefficients × expression level of FRG mRNA. FRGscore of each early-stage LUAD patient in TCGA training cohort and the three validation cohorts was calculated based on the formula. The surv-cutpoint function in “survival” R packages was utilized to stratify early-stage LUAD patients into the FRGscore-high and FRGscore-low subgroups.

### Immune Landscape Analysis in Ferroptosis-Related Gene Score-Defined Subgroups

Infiltration levels of 22 immune cells in individual early-stage LUAD were determined using “CIBERSORT” R package based on LM22 signatures with 1,000 permutations (23). Samples with CIBERSORT *p*-value <0.05 were enrolled for the differential analysis of the immune cell infiltration level between subgroups. The ESTIMATE algorithm was utilized to calculate the immunoscore of individual tumors (24). Moreover, tumor immune estimation resource (TIMER) (https://cistrome.shinyapps.io/timer/) was used to estimate the infiltration level of six immune cells obtained from TCGA dataset (including B cells, CD4+ T cells, CD8+ T cells, neutrophils, macrophages, and dendritic cells). Tumor Immune Dysfunction and Exclusion (TIDE) webserver (http://tide.dfci.harvard.edu) was employed to predict the response to immunotherapy of each sample based on the transcriptome profile. Individual TIDE scores were pooled to predict the efficacy of ICI-based therapy for the FRGscore-high and FRGscore-low subgroups. A higher TIDE score indicates that tumor cells are more likely to undergo immune escape, implying a lower response rate to ICI treatments. Immunophenoscore (IPS) was a predictor of response to anti-PD-1 immunotherapy, which quantifies the determinants of tumor immunogenicity and profiles intratumor immune landscapes and cancer antigenomes (25). The sum of the weighted average Z-score in the four categories was integrated and referred to as the IPS. In addition, six immune subtypes were matched with enrolled early-stage LUAD samples in TCGA cohort (26).

Gene set enrichment analysis (GSEA; http://software.broadinstitute.org/gsea/) was performed using the Hallmark gene set “h.all.v6.2.symbols.gmt” in MSigDB to elucidate the biological signaling pathways associated with differentially expressed genes between the FRGscore-high and FRGscore-low subgroups. Gene set permutations for each parameter were set at 1,000 times to obtain the enrichment score. False discovery rate (FDR) <0.05 and normalized enrichment score (NES) were utilized to identify significantly enriched pathways among different subgroups. Furthermore, genetic alterations of 11 FRG signatures were determined using CBioPortal for cancer genomics (http://www.cbioportal.org) to explore the effect of the signatures on recurrence in early-stage LUAD. The total number of non-synonymous mutations was determined to explore the tumor mutational burden (TMB). Somatic alterations of driver genes were evaluated separately in the FRGscore-high and FRGscore-low subgroups. The “maftool” R package was employed to identify driver genes of early-stage LUAD (27). The top 20 driver genes with the highest frequency of mutation were further identified.

### Collection of Cohorts With Immune Checkpoint Inhibitor-Based Immunotherapy

The prediction value of FRGscore on the efficacy of immunotherapy was estimated in two cohorts with anti-PD-1/PD-L1 therapy. Two publicly available cohorts (GSE135222 and GSE78220) were retrieved (28, 29). Expression profiles and corresponding survival data of 27 lung cancer cases receiving anti-PD-1/PD-L1 immunotherapy and 28 malignant melanoma cases with anti-PD-1 immunotherapy were derived from the GEO database (https://www.ncbi.nlm.nih.gov/geo/).

### Statistical Analysis

All statistical data analyses were performed using R software version 4.0.3 (https://www.R-project.org) and GraphPad Prism version 8.0. Statistical differences were determined using the Wilcoxon test (for two groups) or Kruskal–Wallis test (for more than two groups). Univariate and multivariate Cox regression models were employed to explore the independent prognostic value of FRGscore. Kaplan–Meier method was utilized to generate survival curves using “Survminer” R package. The differences in RFS and OS between subgroups were determined using a log-rank test. Prediction performances of FRG signatures at 1-, 3-, and 5-year RFS and OS were estimated using time-dependent receiver operating characteristic (ROC) curves. The associations between FRGscore and TMB, and FRG-driven signatures were evaluated through Spearman’s correlation analysis. A prognostic meta-analysis was performed using the R package meta to explore the comprehensive prognostic value of FRGscore for different populations in the four cohorts. The random-effects model was applied to calculate the pooled hazard ratio (HR). *p* < 0.05 suggested statistical significance.

## Results

### Distinct Ferroptosis-Defined Patterns Are Significantly Associated With Prognosis and Immune Characteristics

**Figure 1:**
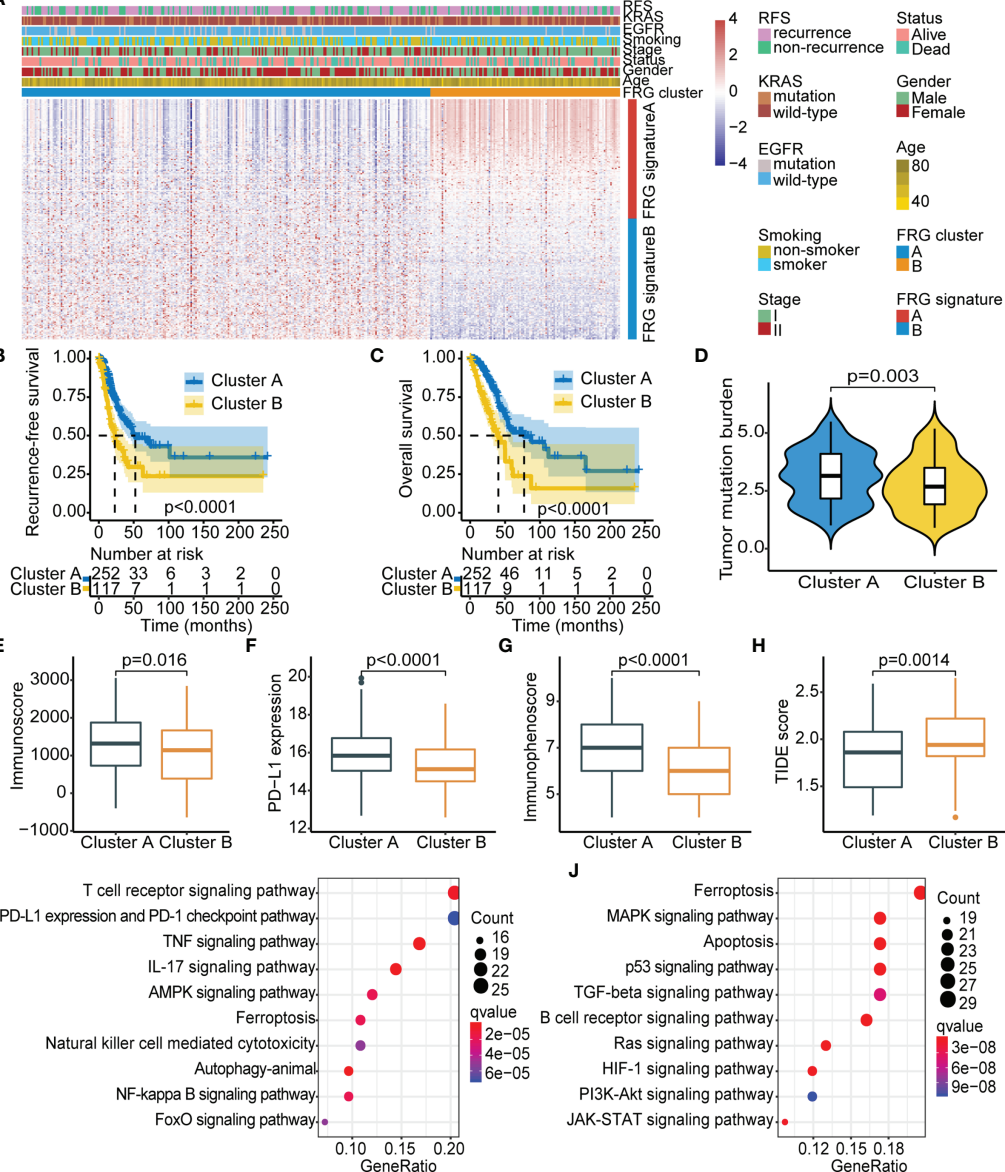
Identification of FRG-based subtypes in early-stage LUAD. **(A)** Unsupervised clustering of FRGs in TCGA cohort. Clinicopathological parameters, including age, gender, stage, smoking status, EGFR/KRAS mutation status, and FRG cluster are shown. Rows represent FRGs, and columns represent samples. **(B)** Kaplan–Meier curves showing recurrence-free survival in the two FRG-based clusters. **(C)** Kaplan–Meier curves showing overall survival in the two FRG-based clusters. **(D–H)** Comparison of TMB **(D)**, immunoscore **(E)**, PD-L1 expression **(F)**, immunophenoscore **(G)**, and TIDE score **(H)** between the two FRG clusters. **(I, J)** KEGG analysis of the two FRG cluster-related signatures: FRG signature A **(I)** and FRG signature B **(J)**. FRG, ferroptosis-related gene; LUAD, lung adenocarcinoma; TCGA, The Cancer Genome Atlas; EGFR, epidermal growth factor receptor; TMB, tumor mutational burden; TIDE, Tumor Immune Dysfunction and Exclusion; KEGG, Kyoto Encyclopedia of Genes and Genomes.

To explore the effect of ferroptosis in early-stage LUAD, unsupervised clustering analysis was performed using ConsesusClusterPlus package to stratify cases with different ferroptosis patterns based on the expression level of 217 FRGs. Notably, two independent cluster subtypes were identified in TCGA cohort, termed FRG cluster A and cluster B (**Figure 1A**). The transcriptome profile of the selected 217 FRGs is presented as a heatmap (**Figure 1A**). The signatures positively related to the FRG-based cluster were defined as FRG signature A, and the other signatures were referred to as FRG signature B (**Table S3**). Analysis showed that FRG cluster A exhibited better RFS and OS than did FRG cluster B (*p* < 0.0001, log-rank test; **Figures 1B, C**). Further, clustering analysis was conducted using the GSE31210 cohort (**Figure S2A**). Consistently, significant differences in RFS and OS were observed between the two subtypes determined by consensus clustering (*p* < 0.0001, log-rank test; **Figures S2B, C**).

To explore the inherent biological differences associated with distinct clinical phenotypes, immune-related parameters of the tumor microenvironment were compared among subgroups. The CIBERSORT and ESTIMATE algorithms were used to determine activity and infiltrating levels of immune cells in tumor tissues and to further characterize the immune infiltration profile. The results showed significantly high infiltration levels of resting dendritic cells, M1 macrophages, activated memory CD4 T cells, activated NK cells, and CD8 T cells in FRG cluster A, whereas FRG cluster B was characterized by high infiltration levels of memory B cells, naive B cells, plasma cells, and M2 macrophages (**Figure S3**). Previous studies suggested that the “immune-exhausted” phenotype presented a gradual increase in matrix component infiltration and M2 macrophages associated with immune suppression and decreased dendritic cell infiltration (30, 31). This implies that low levels of infiltrating immune cells and the abundant matrix components in FRG cluster B may play a role in the inhibition of effective antitumor immune response. Further analysis showed that FRG cluster A exhibited significantly higher TMB, immunoscore, PD-L1 expression, and IPS (**Figures 1D–G**) and lower TIDE score as compared with FRG cluster B (**Figure 1H**). This finding implies that the worse prognosis in FRG cluster B was attributed to tumor immune escape.

Further, KEGG analysis was carried out based on clustering subtypes to explore differences in potential molecular biological characteristics of distinct ferroptosis-defined patterns. The top 10 significant enrichment pathways of FRG signature A and FRG signature B were then identified (**Figures 1I, J**). Notably, FRG cluster A showed significant enrichment in pathways implicated in active immune response, including T-cell receptor signaling pathway and PD-L1 expression and PD-1 checkpoint pathway in cancer. Pathways enriched in FRG cluster B were mainly correlated with oncogenic activation and stromal pathways, including the TGF-β signaling pathway and p53 signaling pathway. These results indicate that FRG cluster A was an immune-inflamed phenotype that harbored immune activation and abundant immune cell infiltration, whereas FRG cluster B was an immune-exhausted phenotype with immune suppression. These results emphasized that the two ferroptosis patterns with different prognoses were characterized by different immune characteristics. In summary, consistency in immune and prognostic profiles of different FRG cluster subtypes indicated that the classification method used was accurate.

### The Ferroptosis-Related Gene Score Effectively Predicts Recurrence in Early-Stage Lung Adenocarcinoma

**Figure 2:**
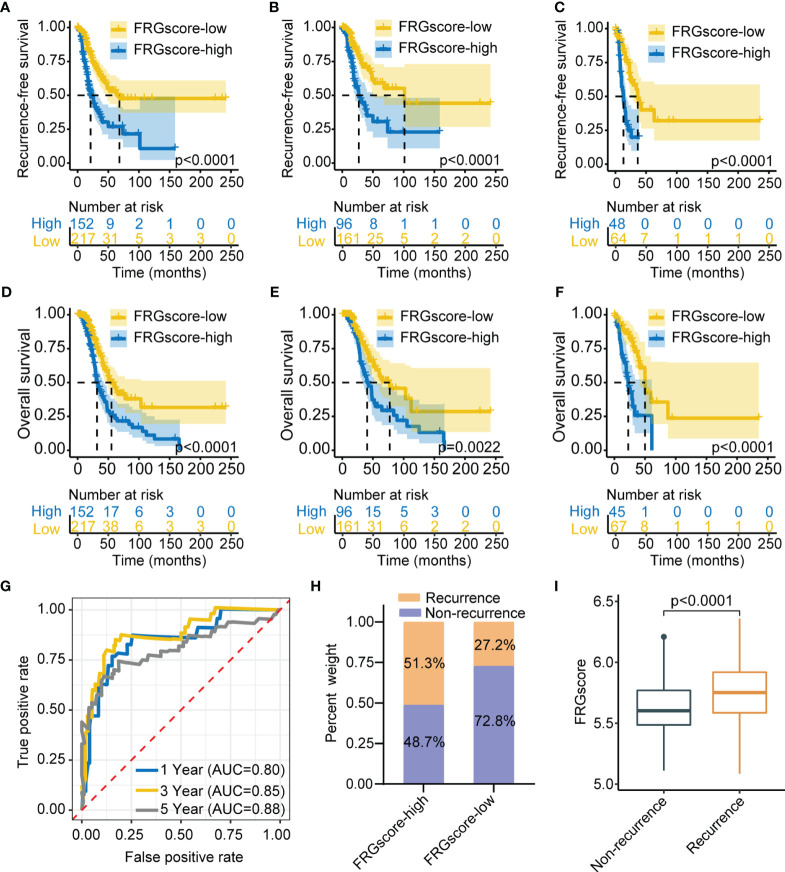
Construction of FRGscore to determine early-stage LUAD recurrence in TCGA cohort. **(A)** Kaplan–Meier curves showing recurrence-free survival in FRGscore-high and FRGscore-low subgroups. **(B, C)** Kaplan–Meier curves showing recurrence-free survival in stage I **(B)** and stage II **(C)** LUAD patients based on FRGscore. **(D)** Kaplan–Meier curves showing OS in FRGscore-high and FRGscore-low subgroups. **(E, F)** Kaplan–Meier curves showing OS in stage I **(E)** and stage II **(F)** LUAD based on FRGscore. **(G)** Time-dependent ROC analysis of FRG signatures for predicting recurrence risk at 1, 3, and 5 years. **(H)** Proportion of recurrence risk in FRGscore-high or FRGscore-low subgroups. **(I)** Distribution of FRGscore in recurrence and non-recurrence subgroups. FRGscore, ferroptosis-related gene score; LUAD, lung adenocarcinoma; TCGA, The Cancer Genome Atlas; OS, overall survival; ROC, receiver operating characteristic.

The previous results showed different FRG-based ferroptosis patterns in early-stage LUAD. Notably, these results were exclusively population-based analyses and could not accurately predict the relationships between FRGs and recurrence and quantify ferroptosis-mediated recurrence patterns of individual patients. Further analysis was thus performed to identify the relationship between FRG and recurrence in early-stage LUAD (**Figure S1**). Univariate Cox model was utilized to assess the association between FRG expression and RFS, Lasso-penalized Cox regression analysis was applied for variable selection, and a stepwise Cox regression model was employed to optimize the established model. A scoring system, termed as FRGscore, was then developed based on identified FRG signatures associated with recurrence. Afterwards, 11 FRG signatures with the highest predictive value for recurrence were determined. The following scoring formula was obtained based on the linear combination of regression coefficient obtained by Cox model and expression levels of FRG signatures: FRGscore = (0.0281 × expression of EIF2S1) − (0.0351 × expression of CHMP6) + (0.0475 × expression of ATF4) + (0.0809 × expression of TFRC) − (0.0071 × expression of DUOX1) + (0.0008 × expression of PSAT1) + (0.0237 × expression of ATG5) + (0.1406 × expression of YY1AP1) − (0.1593 × expression of CAPG) + (0.0232 × expression of IDH1) + (0.1657 × expression of SLC1A5). FRGscore of each early-stage LUAD case was obtained using the scoring formula. The patients in TCGA training set were classified to the FRGscore-high subgroup and FRGscore-low subgroup based on the cutoff value obtained using “survminer” R package. The patients with low FRGscore exhibited significantly longer RFS than did FRGscore-high patients (*p* < 0.0001, log-rank test; **Figure 2A**). Restriction of FRGscore to LUAD patients with stage I showed significant differences in RFS between the FRGscore-high and FRGscore-low subgroups (*p* < 0.0001, log-rank test; **Figure 2B**). Moreover, low FRGscore was correlated with favorable RFS in LUAD patients with stage II (*p* < 0.0001, log-rank test; **Figure 2C**). Analysis of OS indicated that the mortality rate of FRGscore-low patients was evidently lower than that of FRGscore-high patients, after stratification by stage I–II, stage I, and stage II (**Figures 2D–F**). The area under the time-dependent ROC curve was calculated to determine the prediction ability of FRGscore on RFS. The area under the curve (AUC) values of FRGscore based on 11 FRG signatures in predicting recurrence at 1, 3, and 5 years were 0.80, 0.85, and 0.88, respectively (**Figure 2G**). Analyses of the proportion of recurrence and non-recurrence in the FRGscore-high or FRGscore-low subgroup showed a higher number of relapsed patients in the FRGscore-low subgroup, whereas the FRGscore-low subgroup showed a higher number of relapse-free patients (**Figure 2H**). Additionally, a lower FRGscore was observed in relapse-free patients compared with relapsed patients (*p* < 0.0001, Wilcoxon test; **Figure 2I**).

### Validation of the Ferroptosis-Related Gene Score in Three Independent Cohorts

**Figure 3:**
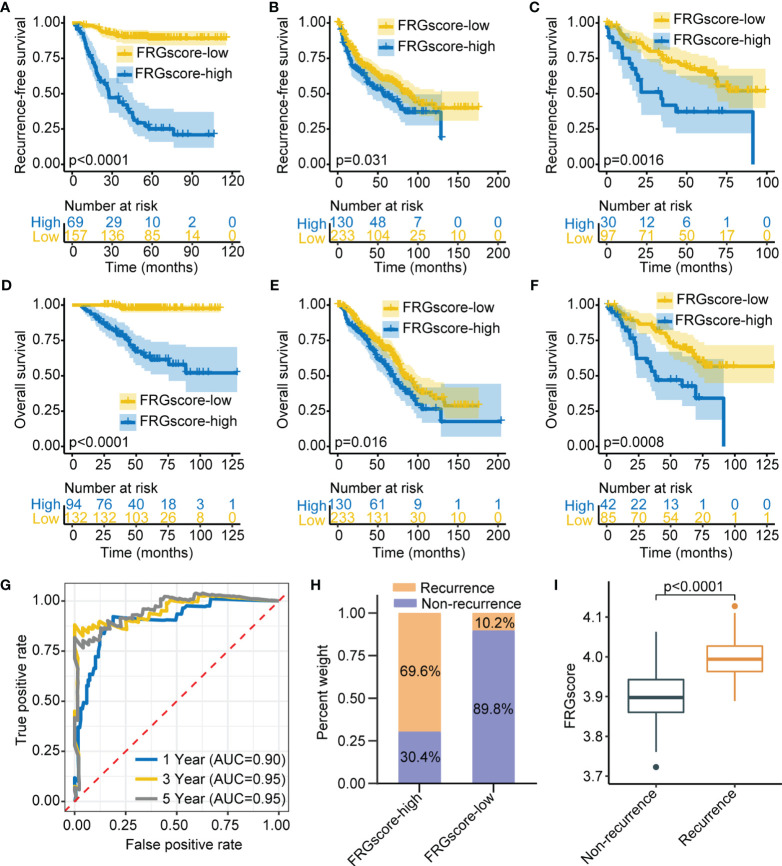
Validation of FRGscore in three independent cohorts. (A–C) Kaplan–Meier curves of recurrence-free survival between FRGscore-high versus FRGscore-low subgroups in GSE31210 cohort **(A)**, GSE68465 cohort **(B)**, and GSE50081 cohort **(C)**. (D–F) Kaplan–Meier curves of OS between FRGscore-high versus FRGscore-low subgroups in GSE31210 cohort **(D)**, GSE68465 cohort **(E)**, and GSE50081 cohort **(F)**. **(G)** Time-dependent ROC analysis based on FRGscore for predicting recurrence risk at 1, 3, and 5 years in GSE31210 cohort. **(H)** Proportion of recurrence risk in FRGscore-high or FRGscore-low subgroups in GSE31210 cohort. **(I)** Difference in FRGscore between recurrence and non-recurrence subgroups in GSE31210 cohort. FRGscore, ferroptosis-related gene score; OS, overall survival; ROC, receiver operating characteristic.

Three independent cohorts (GSE31210, GSE68465, and GSE50081) were used as validation sets to further verify the prediction value of the FRGscore. The corresponding demographic data are presented in **Table S1**. The same scoring scheme described above was used to calculate individual FRGscore; then patients were assigned to the FRGscore-high subgroup and FRGscore-low subgroup. The FRGscore-low subgroup in the GSE31210 cohort showed significantly higher RFS than did the FRGscore-high subgroup (*p* < 0.0001, log-rank test; **Figure 3A**). The RFS of the FRGscore-high subgroup in the GSE68465 cohort was significantly shorter than that in the FRGscore-low subgroup (*p* = 0.031, log-rank test; **Figure 3B**). Higher FRGscore was correlated with worse RFS in the GSE50081 cohort (*p* = 0.0016, log-rank test; **Figure 3C**). Analysis of OS in the three validation cohorts indicated significantly longer survival for the FRGscore-low subgroup over the FRGscore-high subgroup (all *p* < 0.05, log-rank test; **Figures 3D–F**). Moreover, FRGscore of stage I or stage II patients in the GSE31210 cohort accurately distinguished RFS of the FRGscore-high and FRGscore-low subgroups (all *p* < 0.005, log-rank test; **Figure S4A, B**). Analysis of stage I patients in the GSE31210 cohort showed that lower FRGscore was associated with longer OS (*p* < 0.0001, log-rank test; **Figure S4C**). However, analysis of OS in stage II patients showed that the FRGscore was not significantly different between the two subgroups, which might be attributed to the limited sample size (*p* = 0.072, log-rank test; **Figure S4D**). Analysis of the GSE31210 cohort showed that the AUC values of FRGscore in predicting recurrence at 1, 3, and 5 years were 0.90, 0.95, and 0.95, respectively (**Figure 3G**). Notably, the FRGscore-high subgroup mainly comprised relapsed patients, whereas the FRGscore-low subgroup mainly comprised relapse-free patients (**Figure 3H**). Higher FRGscore was mainly observed in relapsed patients (*p* < 0.0001, Wilcoxon test; **Figure 3I**).

### Validation of Ferroptosis-Related Gene Score in Different Clinical Subgroups and Immune Subtypes

**Figure 4:**
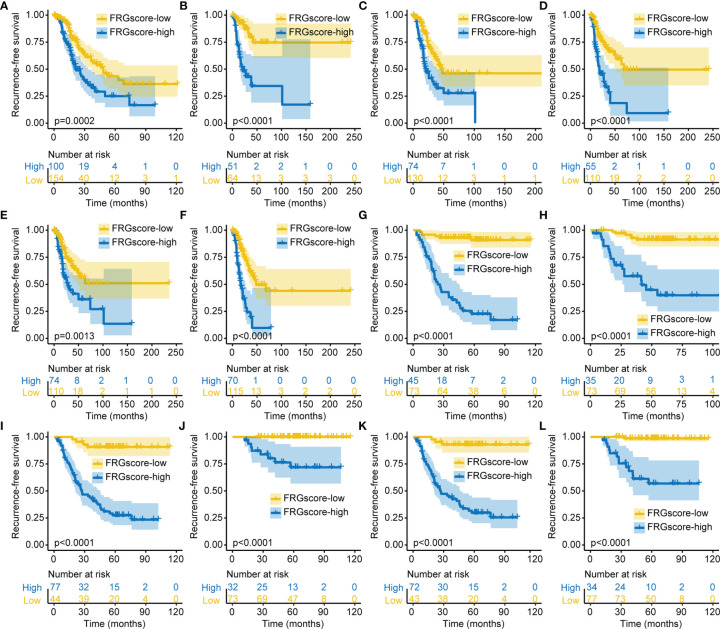
Validation of FRGscore in different clinical subgroups. (A–F) Kaplan–Meier curves of recurrence-free survival in older **(A)**, younger **(B)**, female **(C)**, male **(D)**, non-smoker **(E)**, and smoker **(F)** subgroups for early-stage LUAD patients based on FRGscore in TCGA cohort. **(G–L)** Kaplan–Meier curves of recurrence-free survival in older **(G)**, younger **(H)**, female **(I)**, male **(J)**, non-smoker **(K)**, and smoker **(L)** subgroups for early-stage LUAD patients based on FRGscore in GSE31210 cohort. FRGscore, ferroptosis-related gene score; LUAD, lung adenocarcinoma; TCGA, The Cancer Genome Atlas.

Previous studies demonstrated that gender, age, and smoking were the main risk factors for developing LUAD (32, 33). Therefore, patients with early-stage LUAD were stratified into different subgroups based on the three clinical parameters, including older (age ≥ 60 years), younger (age < 60 years), female, male, non-smokers, and smokers. Analysis of all subgroups for TCGA cohort and GSE31210 cohort showed that FRGscore-low patients exhibited an obvious RFS advantage over FRGscore-high patients (all *p* < 0.005, log-rank test; **Figure 4**). Furthermore, the FRGscore-high subgroup witnessed a shorter OS than did the FRGscore-low subgroup in TCGA and GSE31210 cohorts (all *p* < 0.05, log-rank test; **Figure S5**).

The Pan-Cancer Atlas comprises six immune subtypes (C1–C6) covering multiple cancer types including lung cancer, which represents the functional classification of the tumor microenvironment and serves as an immune response model for prognosis (26). The six immune subtypes include C1 (wound healing), C2 (IFN-γ dominant), C3 (inflammatory), C4 (lymphocyte depleted), C5 (immunologically quiet), and C6 (TGF-β dominant). The distributions of immune subtypes in TCGA cohort mainly comprised C1–3 immune subtypes, with a small number of C4 and C6 cases. The patients were categorized into the FRGscore-high and FRGscore-low subgroups based on FRGscore in the predominant C1–C3 immune subtypes. Analysis of the three immune subtypes (C1–C3) showed that FRGscore-low patients exhibited a prominent RFS and OS benefit as compared with FRGscore-high patients (all *p* < 0.05, log-rank test; **Figures S6A–F**). Furthermore, significantly higher FRGscore was observed for the C1 immune subtype (**Figure S6G**).

**Figure 5:**
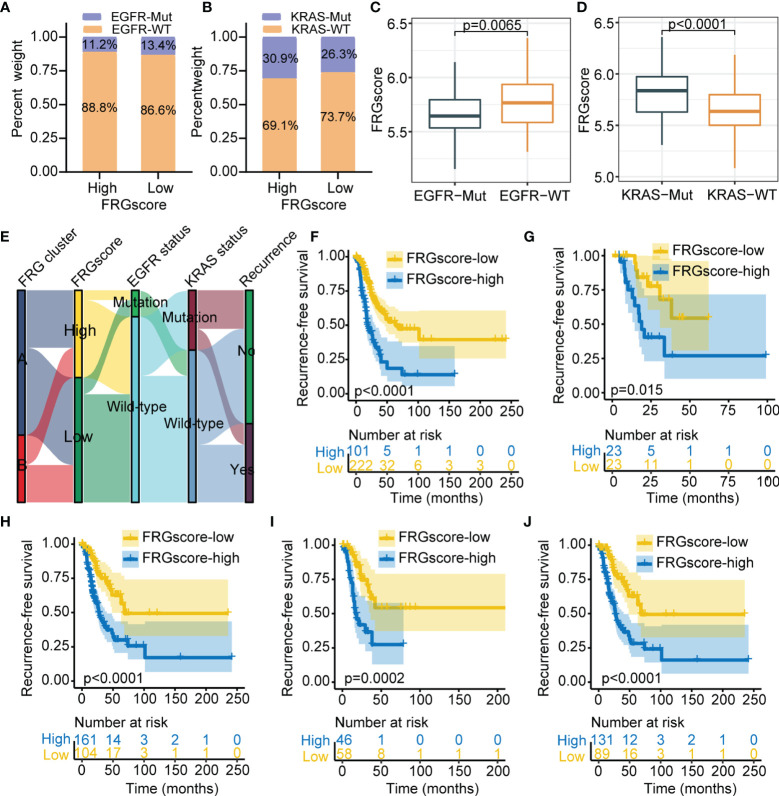
Validation of FRGscore in different molecular subtypes in TCGA cohort. **(A, B)** Proportion of patients harboring EGFR **(A)** and KRAS **(B)** mutations in FRGscore-high or FRGscore-low subgroups. **(C, D)** Distribution of FRGscore in different mutation status of EGFR **(C)** and KRAS **(D)**. **(E)** Alluvial diagram of FRG clusters in groups with FRGscore subgroups, EGFR mutation status, KRAS mutation status, and recurrence status. **(F–J)** Kaplan–Meier curves of recurrence-free survival of patients with EGFR-WT **(F)**, EGFR-Mut **(G)**, KRAS-WT **(H)**, KRAS-Mut **(I)**, and EGFR/KRAS-WT **(J)** based on FRGscore. FRGscore, ferroptosis-related gene score; TCGA, The Cancer Genome Atlas; EGFR, epidermal growth factor receptor.

Previous studies have indicated that driver genes such as EGFR and KRAS were implicated in a distinct immune microenvironment in LUAD (34, 35). Therefore, further analysis was performed to explore the FRGscore profile in different EGFR or KRAS mutation status. Analysis indicated that the proportion of EGFR wild-type (EGFR-WT) was higher compared with the level of EGFR mutant (EGFR-Mut) in the FRGscore-high and FRGscore-low subgroups (**Figure 5A**). Similar results were observed for KRAS parameters (**Figure 5B**). EGFR-Mut subgroup exhibited significantly lower FRGscore over the EGFR-WT subgroup (*p* = 0.0065, Wilcoxon test; **Figure 5C**). Conversely, the KRAS mutant (KRAS-Mut) subgroup showed a higher FRGscore compared with that of the KRAS wild-type (KRAS-WT) subgroup (*p* < 0.0001, Wilcoxon test; **Figure 5D**). Distributions of FRGscore and FRG cluster, EGFR mutation, and KRAS mutation are presented in **Figure 5E**. Analysis of different mutation status including EGFR-WT, EGFR-Mut, KRAS-WT, KRAS-Mut, and EGFR/KRAS-WT showed significantly longer RFS and OS in the FRGscore-low subgroup compared with the FRGscore-high subgroup (all *p* < 0.05, log-rank test; **Figures 5F–J**, **S7**). The results were validated using the GSE31210 cohort (**Figures S8**, **S9**).

The prognostic significance of FRGscore was validated in three independent sets across different clinical subgroups and immune subtypes. The independent prognostic value of FRGscore was further explored in early-stage LUAD. Univariate and multivariate Cox regression analyses were performed based on various clinical parameters including age, gender, stage, smoking history, EGFR, and KRAS mutation status. The results suggested that FRGscore was an independent predictor of RFS and OS after adjusting for clinical parameters in TCGA cohort (**Figures S10A–D**). In addition, the predictive value of FRGscore for RFS and OS was independent of that of other clinical parameters in the GSE31210 cohort (**Figures S10E–H**). A prognostic meta-analysis was conducted to explore the integrated prognostic value of FRGscore in the four cohorts. The results indicated that FRGscore was a significant risk factor for tumor recurrence in patients with early-stage LUAD (combined HR = 2.03, 95% CI =1.49–2.76, *p* < 0.001, random-effects model; **Figure S11**).

### Ferroptosis-Related Gene Score Was Characterized by Distinct Immune Landscapes

**Figure 6:**
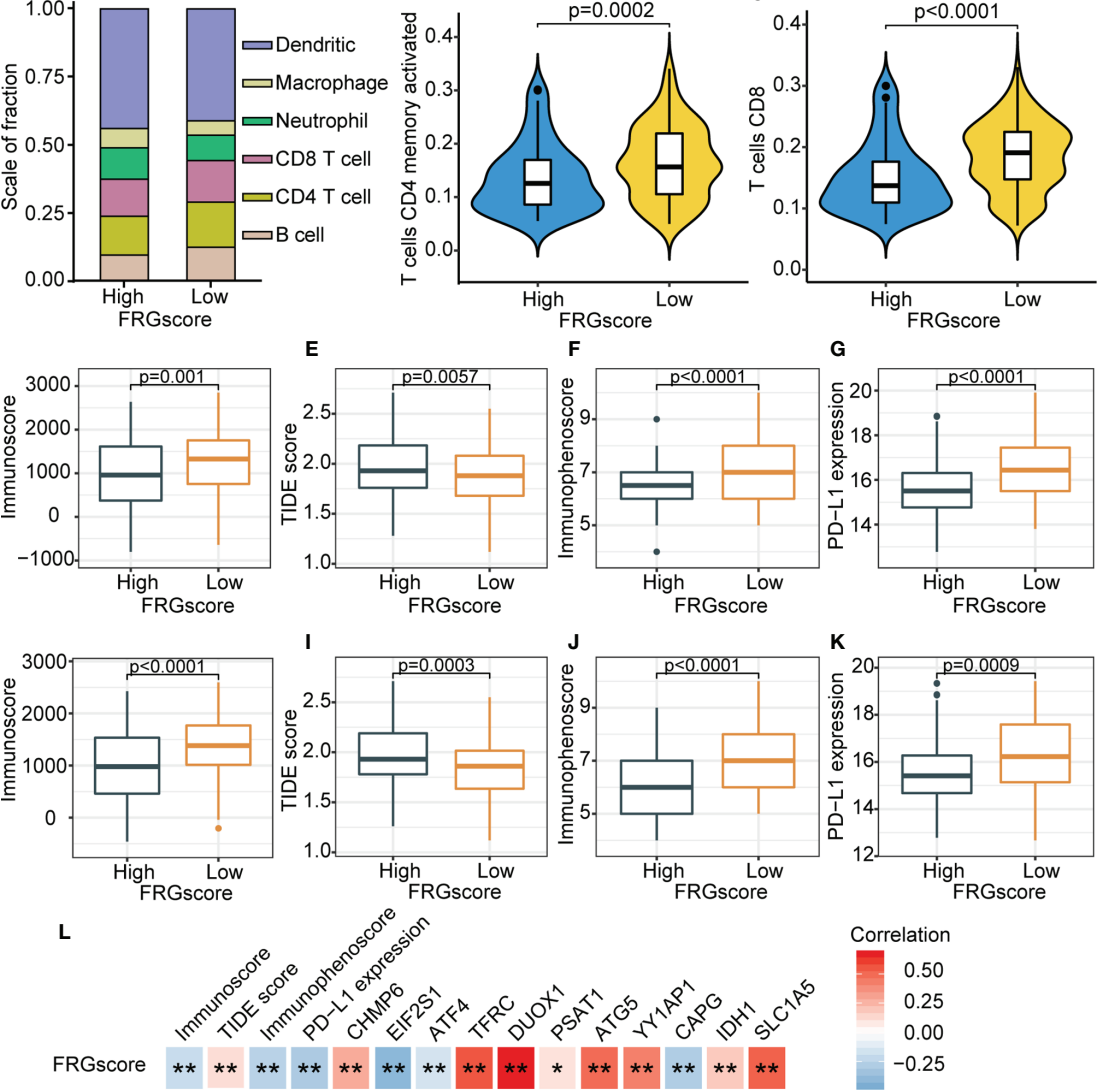
Immune landscape based on FRGscore in early-stage LUAD. **(A)** Composition of infiltrating immune cells in FRGscore-high and FRGscore-low subgroups. **(B, C)** Infiltration levels of CD4 memory-activated T cells **(B)** and CD8 T cells **(C)** in FRGscore-high and FRGscore-low subgroups. **(D–G)** Distributions of immunoscore **(D)**, TIDE score **(E)**, immunophenoscore **(F)**, and PD-L1 expression level **(G)** in FRGscore-high and FRGscore-low subgroups in TCGA cohort. **(H–K)** Distributions of immunoscore **(H)**, TIDE score **(I)**, immunophenoscore **(J)**, and PD-L1 expression level **(K)** in FRGscore-high and FRGscore-low subgroups in GSE31210 cohort. **(L)** Correlations between FRGscore and immune profiles and FRG signatures. *p < 0.05 and **p < 0.01. FRGscore, ferroptosis-related gene score; LUAD, lung adenocarcinoma; TIDE, Tumor Immune Dysfunction and Exclusion; TCGA, The Cancer Genome Atlas.

The previous results showed that different ferroptosis patterns were associated with distinct prognoses and immune profiles. Therefore, analyses based on an immune profile were performed to explore the underlying mechanism for the relationship between FRG-driven signatures and different recurrence risks. GSEA suggested apoptosis (NES = 1.993, normalized *p* = 0.019), AMPK signaling pathway (NES = 1.833, normalized *p* = 0.037), PI3K-Akt signaling pathway (NES = 2.098, normalized *p* = 0.008), PPAR signaling pathway (NES = 2.197, normalized *p* = 0.005), and NF-kappa B signaling pathway (NES = 2.213, normalized *p* = 0.005) were evidently enriched in the FRGscore-high subgroup (**Figure S12**). Moreover, immune-related pathways, including TNF signaling pathway (NES = −2.379, normalized *p* < 0.001), cell activation involved in immune response (NES = −2.366, normalized *p* < 0.001), immune response regulating signaling pathway (NES = −2.362, normalized *p* < 0.001), T-cell receptor signaling pathway (NES = −2.331, normalized *p* < 0.001), and IL-17 signaling pathway (NES = −2.293, normalized *p* < 0.001), were mainly enriched in the FRGscore-low subgroup (**Figure S12**). Infiltrating levels of the six immune cells between the FRGscore-high and FRGscore-low subgroups were characterized (**Figure 6A**). The results indicated that the FRGscore-low subgroup in TCGA cohort had markedly higher levels of activated memory CD4 T cells and CD8 T cells compared with the levels in the FRGscore-high subgroup (**Figures 6B, C**).

ICI-based therapies represented by CTLA-4/PD-1/PD-L1 inhibitors have significantly revolutionized antitumor treatment. In addition to the well-known TMB, PD-L1, and microsatellite instability (MSI), newly discovered predictors, such as TIDE and IPS, have been widely utilized and highly recommended for the assessment of immune response (36). Further analyses of immune parameters showed that the FRGscore-low subgroup exhibited significantly higher immunoscore, higher IPS and PD-L1 expression levels, and lower TIDE score than did the FRGscore-high subgroup (**Figures 6D–G**). Similar results were observed for the GSE31210 cohort (**Figures 6H–K**). Analyses of these immune parameters indicated that the unfavorable prognosis in the FRGscore-high subgroup may be related to tumor-mediated immune escape. Further analysis was performed to explore correlations between FRGscore and immune parameters and FRG signatures. The results showed that FRGscore was positively correlated with immunoscore, IPS, and PD-L1 expression levels but negatively correlated with the TIDE score (**Figure 6L**). Analysis of the 11 FRG signatures indicated that FRGscore was significantly negatively correlated with EIF2S1, ATF4, and CAPG and showed a significantly positive correlation with the other signatures (**Figure 6L**). These findings imply that FRG-driven signatures exert a significant role in ferroptosis-mediated immune response. Consequently, the potential prognostic value of FRGscore might be attributed to the favorable immune response and less aggressive tumor growth.

**Figure 7:**
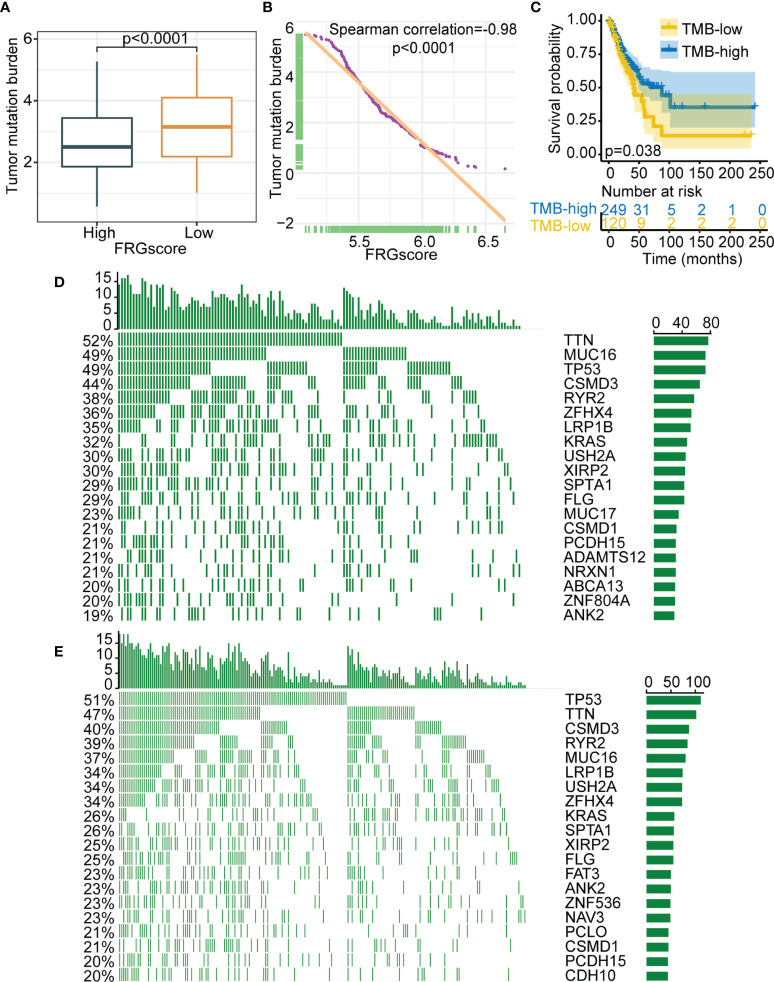
Relationship between FRGscore and somatic variants. **(A)** Distribution of TMB in FRGscore-high and FRGscore-low subgroups. **(B)** Spearman’s correlation between FRGscore and TMB in TCGA cohort. **(C)** Kaplan–Meier curves of TMB-high and TMB-low subgroups in TCGA cohort. **(D, E)** Distributions of top 20 most frequently mutated genes in FRGscore-high subgroup **(D)** and FRGscore-low subgroup **(E)**. FRGscore, ferroptosis-related gene score; TMB, tumor mutational burden; TCGA, The Cancer Genome Atlas.

Accumulating evidence suggests that tumor genomic somatic mutations are correlated with immunotherapy response. Thus, the distribution pattern of TMB was analyzed to gain insight into the immunological properties of different FRGscore subgroups. The results demonstrated that the FRGscore-low subgroup had higher TMB than had the FRGscore-high subgroup (*p* < 0.0001, Wilcoxon test; **Figure 7A**). Correlation analysis suggested that FRGscore was significantly negatively associated with TMB (**Figure 7B**). TMB-high patients had significantly longer survival than TMB-low patients (*p* = 0.038, log-rank test; **Figure 7C**). Analysis of significantly mutated genes between the FRGscore-high and FRGscore-low subgroups in early-stage LUAD was performed. The mutation profile indicated that the FRGscore-high subgroup harbored higher somatic mutation rates of MUC16 (49% vs. 39%) and KRAS (32% vs. 26%) than did the FRGscore-low subgroup (**Figures 7D–E**). Moreover, analysis of genetic alterations of the 11 FRG signatures in TCGA cohort suggested that YY1AP1 and TFRC were the top two signatures with the highest mutation frequency, with mutation rates of 32% and 14%, respectively (**Figure S13**). These findings provide a basis for comprehensive characterization of the role of FRGscore-defined classification on genomic variation and show potentially complex interactions between individual somatic mutations and FRGscore.

### Role of Ferroptosis-Related Gene Score in Predicting Immunotherapeutic Response

**Figure 8:**
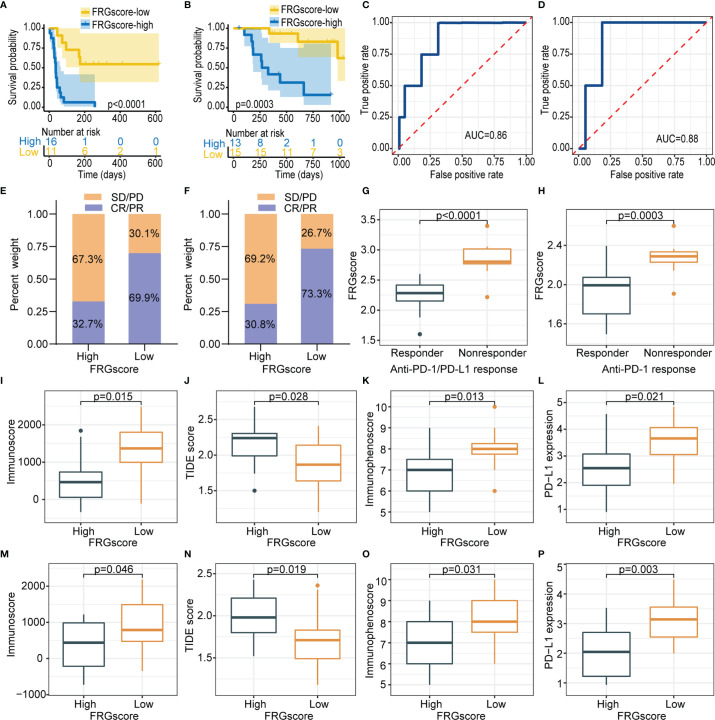
FRGscore is an effective predictor of immunotherapeutic benefits. **(A, B)** Kaplan–Meier curves of GSE135222 cohort **(A)** and GSE78220 cohort **(B)** in FRGscore-high and FRGscore-low subgroups. **(C, D)** ROC curve of FRGscore in predicting immunotherapeutic response in GSE135222 cohort **(C)** and GSE78220 cohort **(D)**. **(E, F)** Proportion of patients with positive clinical response to anti-PD-1/PD-L1 immunotherapy in FRGscore-high or FRGscore-low subgroups in GSE135222 cohort **(E)** and GSE78220 cohort **(F)**. **(G, H)** Distributions of FRGscore in distinct response status to anti-PD-1/PD-L1 immunotherapy in GSE135222 cohort **(G)** and GSE78220 cohort **(H)**. (I–L) Distributions of immunoscore **(I)**, TIDE score **(J)**, immunophenoscore **(K)**, and PD-L1 expression level **(L)** in FRGscore-high and FRGscore-low subgroups in GSE135222 cohort. **(M–P)** Distributions of immunoscore **(M)**, TIDE score **(N)**, immunophenoscore **(O)**, and PD-L1 expression level **(P)** in FRGscore-high and FRGscore-low subgroups in GSE78220 cohort. FRGscore, ferroptosis-related gene score; ROC, receiver operating characteristic; TIDE, Tumor Immune Dysfunction and Exclusion.

The role of FRGscore in predicting the response to ICI-based therapy was explored in two independent immunotherapy cohorts owing to the significant correlation between FRGscore and immune response. The patients receiving immunotherapy in GSE135222 (anti-PD-1/PD-L1 immunotherapy) and GSE78220 (anti-PD-1 immunotherapy) cohorts were assigned to the FRGscore-high and FRGscore-low subgroups. The patients with low FRGscore in the two cohorts exhibited favorable clinical advantages and significantly longer survival over FRGscore-high patients (all *p* < 0.0005, log-rank test; **Figures 8A, B**). AUC values of FRGscore in predicting survival in the GSE135222 and GSE78220 cohorts were 0.86 and 0.88, respectively (**Figures 8C, D**). These results implied that FRGscore distinguished different clinical outcomes of patients with anti-PD-1/PD-L1 immunotherapy.

Distributions of treatment response rates between FRGscore-high and FRGscore-low patients further showed significant therapeutic benefit and immune response of ICI-based therapy (response rate of anti-PD-1/PD-L1 cohort: 69.9% vs. 32.7%, **Figure 8E**; response rate of anti-PD-1 cohort: 73.3% vs. 30.8%, **Figure 8F**). The results demonstrated lower FRGscore in patients who responded to anti-PD-1/PD-L1 immunotherapy (**Figures 8G, H**). Moreover, immune parameters between different FRGscore subgroups were analyzed based on two immunotherapy cohorts. Immunoscore, IPS, and PD-L1 expression levels were evidently higher in the FRGscore-low subgroup in the GSE135222 cohort, whereas the TIDE score was significantly decreased compared with that in the FRGscore-high subgroup (**Figures 8I–L**). Consistent results were obtained for the GSE78220 cohort (**Figures 8M–P**). Collectively, the results indicated that FRGscore was associated with response to immunotherapy and was a predictor for immunotherapy prognosis of LUAD patients.

## Discussion

The prevalence of early-stage cases has significantly increased owing to the increasing use of low-dose CT (LDCT) for lung cancer screening in high-risk groups (37). Optimizing treatment strategies and clinical management of early-stage diseases is therefore required to improve the prognosis of lung cancer. This highlights the urgent need for the identification of robust biomarkers for early-stage individuals presenting with high-risk tumor relapse who mostly benefit from additional systemic treatments. Given that ferroptosis was highly correlated with the occurrence and progression of LUAD (11), development of ferroptosis-related recurrence signatures in early-stage LUAD is considered to be a priority. Therefore, the present study sought to explore the association between FRGs and early-stage LUAD recurrence. Two different ferroptosis-based patterns were determined, which effectively distinguished prognostic profiling and immune characteristics in different subgroups. A total of 11 FRG-based signature panels significantly related to RFS were identified and validated in early-stage LUAD patients using large-scale datasets from multi-cohorts. A scoring scheme constructed based on the 11 FRG signatures panel effectively distinguished recurrence patterns, and its reliability was verified using different clinical subgroups and immune subtypes. Furthermore, the results showed that the FRGscore was an independent prognostic indicator for early-stage LUAD. Notably, FRGscore-based differences indicated distinct immune landscapes and were correlated with efficacy of ICI therapy.

The present study characterized two ferroptosis-mediated patterns with high accuracy in prognosis prediction and discrimination of different immunophenotypes. FRG cluster A exhibited higher RFS and OS than did FRG cluster B. Previous studies suggested that immune-inflamed phenotype and immune-exhausted phenotype had significant differences in several immune components including M2 macrophages, B cells, and dendritic cells (30, 38). Two immunophenotypes of the ferroptosis-defined patterns showed consistent findings with previous reports. FRG cluster A was characterized by activated immune response and abundant expressions of infiltration immune cells, corresponding to immune-inflamed phenotype. On the contrary, FRG cluster B was characterized by immune-suppressive response, corresponding to immune-exhausted phenotype. Galon et al. reported that the tumor microenvironment exerts a vital role in tumor progression and efficacy of immunotherapy (39). Baseline levels of tumor-infiltrating CD4+/CD8+ T cells, macrophage M1, and inflammatory cytokine secretion were implicated in immune response (40, 41). The results showed that FRG cluster A had significantly longer RFS and OS and exhibited significantly elevated infiltration levels of M1 macrophages, CD4 T cells, and CD8 T cells compared with FRG cluster B. The results were consistent with findings from previous studies that high infiltration levels of T cells and M1 macrophages were correlated with favorable prognoses (42, 43). In addition, the analysis indicated that FRG cluster A was associated with enrichment of immune-active pathways, whereas FRG cluster B showed significant enrichment of tumor-promoting signaling pathways, as reported previously (30). These results implied that FRG cluster A exhibited higher immune response to tumor progression and aggressiveness. Moreover, associations between different ferroptosis patterns and several known predictive biomarkers of immunotherapy such as TMB, PD-L1 expression, and TIDE score were explored (44–46). Notably, the analysis showed that FRG cluster A was significantly correlated with predictive parameters of immunotherapy response and showed significant enrichment of immune activation pathways, implying a potential predictive value of immunotherapy benefits. Identification of tumor immune characteristics of different ferroptosis patterns provides insight into the interactions of ferroptosis with antitumor immune response and a basis for the development of effective immunotherapy strategies.

FRG scoring system based on recurrence-related signatures was further established to quantify different ferroptosis patterns and to accurately guide individualized treatment strategies for early-stage LUAD patients. FRGscore was constructed based on 11 FRG signatures with the predictive value of recurrence in TCGA cohort. The results revealed that FRGscore-low patients had a longer RFS, and patients without LUAD recurrence exhibited a lower FRGscore, which allowed FRGscore to distinguish patients at risk of recurrence. Further, the constructed FRGscore was validated using three independent cohorts. A high prognostic performance confers any signature more attractive for clinical application in imperative subgroups of early-stage LUAD. The results suggested that FRGscore effectively distinguished the risk of recurrence in non-smoker, smoker, older, younger, female, and male subgroups. These results indicated that the FRGscore could be used in identifying early-stage LUAD patients with high-risk recurrence in any clinical subgroup. Early-stage LUAD was characterized by histological variation, driver mutation, and heterogeneity of molecular subtypes (35). Previous studies suggested that EGFR and KRAS mutations were highly correlated with the expression of immune molecules and immune cell infiltration (47). Therefore, the prognostic performance of FRGscore in EGFR-WT, EGFR-Mut, KRAS-WT, KRAS-Mut, and EGFR/KRAS-WT subgroups was explored in the current study. A meta-analysis comprising the four cohorts was conducted to further explore the predictive value of the FRG scoring system in assessing the risk of recurrence in early-stage LUAD. Postoperative recurrence was the key cause of tumor death in early-stage LUAD; thus, the association between FRGscore and OS was evaluated. The results indicated that FRGscore was significantly related to the OS of early-stage LUAD patients. Wang et al. previously established an OS-based FRG signature in LUAD (12). Notably, the identified FRG-related signature in the current study was based on recurrence rather than OS, allowing accurate classification of high-risk patients with early-stage LUAD.

The constructed FRGscore accurately distinguished different recurrence patterns and outcomes of early-stage LUAD across clinical and molecular subgroups. Further, the underlying mechanism on the correlation between FRG-based characteristics with different recurrence patterns was explored. Gao et al. and Zhang et al. showed the interface between ferroptosis-related signatures and LUAD prognosis (17, 18). However, an integrated understanding of FRG-driven signatures in early-stage LUAD, including the interactions between the FRG-driven signatures and immunotherapy response, is lacking. GSEA showed significant enrichment of immune-active pathways in the FRGscore-low subgroup. The FRGscore-high subgroup was characterized by tumor malignant features including PI3K-Akt signaling pathway and NF-κB signaling pathway. Analysis of immune infiltration profiles indicated significantly higher proportions of activated memory CD4 T cells and CD8 T cells in the FRGscore-low subgroup. Moreover, FRGscore was significantly associated with predictors of immunotherapy response, including TMB, PD-L1 expression, IPS, and TIDE score, implying that the ferroptosis-related pathway is correlated with the efficacy of immunotherapy. Activated CD8+ T cells promote ferroptosis-specific lipid peroxidation reactions in intra-tumoral cells during immunotherapy and induce activation of the ferroptosis pathway resulting in the antitumor activity of immunotherapy (15). The robust prediction performance of FRGscore for immune response was further confirmed using two independent immunotherapy cohorts. Previous studies reported that lipid metabolites released by ferroptosis cells exerted an immunomodulatory effect on neighboring immune cells, thus triggering an immune response (48). Therefore, the favorable prognosis of the FRGscore-low subgroup might be attributed to the enhanced antitumor immune response. This finding indicates that variable tumor immune response may affect the progression of early-stage LUAD. Additionally, ferroptosis could promote tumor immune escape by directly interfering with the functions of various immune cells (49). Activity of ferroptosis-mediated immune cells is significantly impaired, thus reducing antitumor immunity and promoting tumor metastasis (48). The unfavorable prognosis of the FRGscore-high subgroup may be correlated with the suppressive function of immune cells, leading to tumor immune escape. Ferroptosis plays a crucial role in antitumor immunity, by promoting the killing of tumor cells by T cells activated by ICI therapy and by directly affecting the functions of multiple immune cells, indicating potential synergistic treatment of cancer (48). Therefore, the difference in prognosis between the FRGscore-high and FRGscore-low subgroups could be attributed to the distinct immune profiles. Moreover, FRGscore-based differences may indicate the efficacy of ICI therapy. These findings further suggest that the ferroptosis-defined pattern might be applied to clinical practice to characterize the immunophenotype and provide a basis for the development of effective immunotherapeutic strategies.

After multiple clinicopathological features in different cohorts were adjusted, the result showed that FRGscore was an independent prognostic factor, indicating that it is a promising ferroptosis-related prognostic biomarker. The current study had a few limitations. Although a literature review was conducted and 217 common FRGs were identified, several newly discovered FRGs should be incorporated into the model to further increase the accuracy of the established ferroptosis model. Correlations between FRGscore and immune cell infiltration were based on evaluated tumor characterization. Moreover, ferroptosis patterns and FRGscore were identified and validated in retrospective datasets. Therefore, a prospective cohort of early-stage LUAD patients with immunotherapy is warranted to further validate the findings of the current study.

## Conclusions

In summary, the present study explored the ferroptosis patterns of 1,085 early-stage LUAD samples based on 217 FRGs and evaluated the association between ferroptosis-mediated patterns and distinct immune characteristics. The present study characterized the development and detection of recurrence-related FRG signatures for early-stage LUAD and provided new insights on the correlation between ferroptosis and early-stage LUAD recurrence. FRGscore-based differences could help distinguish distinct recurrence patterns, immune, and molecular characteristics and predict the prognosis of patients receiving ICI-based therapy. Moreover, FRGscore is a promising tool to help clinicians develop personalized treatment strategies, mainly for the selection of populations that significantly benefit from ICI-based therapy, thus effectively reducing relapse rates of these patients. In addition, the comprehensive analysis showed that ferroptosis provides a basis for understanding tumor immune regulation, and evaluating the ferroptosis-mediated pattern of individual tumors will allow enhancing the understanding of immune characteristics and yield important insights for the development of tailoring immunotherapeutic strategies.

## Data Availability Statement

The original contributions presented in the study are included in the article/**Supplementary Material**. Further inquiries can be directed to the corresponding authors.

## Author Contributions

Drafting of the manuscript: LY and PH. Study concept and design: LY and GW. Acquisition, analysis, or interpretation of data: LY, GW, LG, XZ, CW, JZ, DZ, and PH. Critical revision of the manuscript for important intellectual content: LY, GW, PH, XZ, CW, and LG. Statistical analysis: LY, PH, XZ, and LG. Study supervision: LY. All authors read and approved the final manuscript.

## Conflict of Interest

The authors declare that the research was conducted in the absence of any commercial or financial relationships that could be construed as a potential conflict of interest.

## Publisher’s Note

All claims expressed in this article are solely those of the authors and do not necessarily represent those of their affiliated organizations, or those of the publisher, the editors and the reviewers. Any product that may be evaluated in this article, or claim that may be made by its manufacturer, is not guaranteed or endorsed by the publisher.

## Supplementary Material

Supplementary Figure 1 A pipeline for generation and validation of FRG signatures.

Supplementary Figure 2 Validation of FRGs-based subtypes in GSE31210 cohort. **(A)** Unsupervised clustering of FRGs. **(B, C)** Kaplan-Meier curves of recurrence-free survival **(B)** and overall survival **(C)** for early-stage LUAD patients in two FRG-based clusters.

Supplementary Figure 3 Levels of 22 infiltrating immune cells in two FRG-based clusters determined by CIBERSORT algorithm. ns, no significance; *p < 0.05; **p < 0.01.

Supplementary Figure 4 Survival curves of FRGscore in GSE31210 cohort. **(A-B)** Kaplan-Meier recurrence-free survival curves in stage I **(A)** and stage II **(B)** LUAD patients based on FRGscore. **(C, D)** Kaplan-Meier overall survival curves in stage I **(C)** and stage II **(D)** LUAD patients based on FRGscore.

Supplementary Figure 5 Survival analysis of early-stage LUAD patients stratified by age, gender and smoking history. **(A–F)** Kaplan-Meier curves of overall survival in older **(A)**, younger **(B)**, female **(C)**, male **(D)**, non-smokers **(E)** and smokers **(F)** patients based on FRGscore in TCGA cohort. **(G-L)** Kaplan-Meier curves of overall survival in older **(G)**, younger **(H)**, female **(I)**, male **(J)**, non-smokers **(K)** and smokers **(L)** patients based on FRGscore in GSE31210 cohort.

Supplementary Figure 6 Survival analysis of early-stage LUAD patients stratified by immune subtypes in TCGA cohort. **(A–C)** Kaplan-Meier curves of recurrence-free survival in C1 **(A)**, C2 **(B)** and C3 **(C)** immune subtypes based on FRGscore. **(D–F)** Kaplan-Meier curves of overall survival in C1 **(D)**, C2 **(E)** and C3 **(F)** immune subtypes based on FRGscore. **(G)** Distribution of FRGscore in different immune subtypes.

Supplementary Figure 7 Survival analysis of early-stage LUAD patients harboring EGFR or KRAS mutation in TCGA cohort. Kaplan-Meier curves of overall survival in EGFR-WT **(A)**, EGFR-Mut **(B)**, KRAS-WT **(C)**, KRAS-Mut **(D)** and EGFR/KRAS-WT **(E)** patients based on FRGscore.

Supplementary Figure 8 Validation of FRGscore in different molecular subgroups in GSE31210 cohort. **(A, B)** Proportion of patients harboring EGFR **(A)** and KRAS **(B)** mutations in FRGscore-high or FRGscore-low subgroups. **(C–D)** Distribution of FRGscore in different mutation status of EGFR **(C)** and KRAS **(D)**. **(E–I)** Kaplan-Meier curves of recurrence-free survival in EGFR-WT **(E)**, EGFR-Mut **(F)**, KRAS-WT **(G)**, KRAS-Mut **(H)** and EGFR/KRAS-WT **(I)** patients based on FRGscore.

Supplementary Figure 9 Survival analysis of early-stage LUAD patients harboring EGFR or KRAS mutation in GSE31210 cohort. Kaplan-Meier curves of overall survival in EGFR-WT **(A)**, EGFR-Mut **(B)**, KRAS-WT **(C)**, KRAS-Mut **(D)** and EGFR/KRAS-WT **(E)** patients based on FRGscore.

Supplementary Figure 10 Independent prognostic value of FRGscore. **(A, B)** Univariate **(A)** and multivariate **(B)** Cox analyses of recurrence-free survival based on FRGscore in TCGA cohort. **(C–D)** Univariate **(C)** and multivariate **(D)** Cox analyses of overall survival based on FRGscore in TCGA cohort. **(E-F)** Univariate **(E)** and multivariate **(F)** Cox analyses of recurrence-free survival based on FRGscore in GSE31210 cohort. **(G, H)** Univariate **(G)** and multivariate **(H)** Cox analyses of overall survival based on FRGscore in GSE31210 cohort.

Supplementary Figure 11 Meta-analysis of prognostic values of FRG signatures in early-stage LUAD patients across the four cohorts. P-values of TCGA **(n=369)**, GSE31210 **(n=226)**, GSE68465 **(n=363)** and GSE50081 **(n=127)** were calculated by Kaplan-Meier curves. P-value of overall **(n=1085)** patients was calculated by meta-analysis.

Supplementary Figure 12 Biological pathways associated with FRGscore-high and FRGscore-low subgroups.

Supplementary Figure 13 Genetic alteration of the identified 11 FRG signatures in TCGA-LUAD cohort.

Supplementary Table 1 Clinical characteristics of patients with early-stage lung adenocarcinoma in the four datasets.

Supplementary Table 2 Data information used in this study.

Supplementary Table 3 FRG signature genes **(A, B)**.

## Abbreviations

LUAD, early-stage lung adenocarcinoma; FRGscore, ferroptosis-related gene score; ICI, immune checkpoint inhibitor; FRG, ferroptosis-related gene; RFS, recurrence-free survival; OS, overall survival; GEO, Gene Expression Omnibus; TCGA, The Cancer Genome Atlas; KEGG, Kyoto Encyclopedia of Genes and Genomes; TIMER, the tumor immune estimation resource; TIDE, Tumor Immune Dysfunction and Exclusion; IPS, immunophenoscore; GSEA, gene set enrichment analysis; NES, normalized enrichment score; FDR, false discovery rate; TMB, tumor mutational burden; ROC, receiver operating characteristic; HR, hazard ratio; AUC, area under the curve; EGFR-WT, epidermal growth factor receptor wild-type; EGFR-Mut, epidermal growth factor receptor mutant; KRAS-Mut, KRAS mutant; KRAS-WT, KRAS wild-type; LDCT, low-dose CT.

## References

[1] Sung H Ferlay J Siegel RL Laversanne M Soerjomataram I Jemal A. Global Cancer Statistics 2020: GLOBOCAN Estimates of Incidence and Mortality Worldwide for 36 Cancers in 185 Countries. CA: Cancer J Clin (2021) 71(3):209–49. doi: 10.3322/caac.21660 33538338

[2] Abe Y Tanaka N. The Hedgehog Signaling Networks in Lung Cancer: The Mechanisms and Roles in Tumor Progression and Implications for Cancer Therapy. BioMed Res Int (2016) 2016:7969286. doi: 10.1155/2016/7969286 28105432 PMC5220431

[3] Uramoto H Tanaka F. Recurrence After Surgery in Patients With NSCLC. Transl Lung Cancer Res (2014) 3(4):242–9. doi: 10.3978/j.issn.2218-6751.2013.12.05 PMC4367696 25806307

[4] Goss PE Strasser-Weippl K Lee-Bychkovsky BL Fan L Li J Chavarri-Guerra Y. Challenges to Effective Cancer Control in China, India, and Russia. Lancet Oncol (2014) 15(5):489–538. doi: 10.1016/S1470-2045(14)70029-4 24731404

[5] Tanoue LT Tanner NT Gould MK Silvestri GA. Lung Cancer Screening. Am J Respir Crit Care Med (2015) 191(1):19–33. doi: 10.1164/rccm.201410-1777CI 25369325

[6] Stockwell BR Friedmann Angeli JP Bayir H Bush AI Conrad M Dixon SJ. Ferroptosis: A Regulated Cell Death Nexus Linking Metabolism, Redox Biology, and Disease. Cell (2017) 171(2):273–85. doi: 10.1016/j.cell.2017.09.021 PMC5685180 28985560

[7] Mou Y Wang J Wu J He D Zhang C Duan C. Ferroptosis, a New Form of Cell Death: Opportunities and Challenges in Cancer. J Hematol Oncol (2019) 12(1):34. doi: 10.1186/s13045-019-0720-y 30925886 PMC6441206

[8] Lu B Chen XB Ying MD He QJ Cao J Yang B. The Role of Ferroptosis in Cancer Development and Treatment Response. Front Pharmacol (2017) 8:992. doi: 10.3389/fphar.2017.00992 29375387 PMC5770584

[9] Gao M Monian P Quadri N Ramasamy R Jiang X. Glutaminolysis and Transferrin Regulate Ferroptosis. Mol Cell (2015) 59(2):298–308. doi: 10.1016/j.molcel.2015.06.011 26166707 PMC4506736

[10] Lai Y Zhang Z Li J Li W Huang Z Zhang C. STYK1/NOK Correlates With Ferroptosis in Non-Small Cell Lung Carcinoma. Biochem Biophys Res Commun (2019) 519(4):659–66. doi: 10.1016/j.bbrc.2019.09.032 31542233

[11] Alvarez SW Sviderskiy VO Terzi EM Papagiannakopoulos T Moreira AL Adams S. NFS1 Undergoes Positive Selection in Lung Tumours and Protects Cells From Ferroptosis. Nature (2017) 551(7682):639–43. doi: 10.1038/nature24637 PMC5808442 29168506

[12] Wang S Wu C Ma D Hu Q. Identification of a Ferroptosis-Related Gene Signature (FRGS) for Predicting Clinical Outcome in Lung Adenocarcinoma. PeerJ (2021) 9:e11233. doi: 10.7717/peerj.11233 33954048 PMC8051350

[13] Friedmann Angeli JP Krysko DV Conrad M. Ferroptosis at the Crossroads of Cancer-Acquired Drug Resistance and Immune Evasion. Nat Rev Cancer (2019) 19(7):405–14. doi: 10.1038/s41568-019-0149-1 31101865

[14] Hassannia B Vandenabeele P Vanden Berghe T. Targeting Ferroptosis to Iron Out Cancer. Cancer Cell (2019) 35(6):830–49. doi: 10.1016/j.ccell.2019.04.002 31105042

[15] Wang W Green M Choi JE Gijón M Kennedy PD Johnson JK. CD8 T Cells Regulate Tumour Ferroptosis During Cancer Immunotherapy. Nature (2019) 569(7755):270–4. doi: 10.1038/s41586-019-1170-y PMC6533917 31043744

[16] Medina CB Mehrotra P Arandjelovic S Perry JSA Guo Y Morioka S. Metabolites Released From Apoptotic Cells Act as Tissue Messengers. Nature (2020) 580(7801):130–5. doi: 10.1038/s41586-020-2121-3 PMC7217709 32238926

[17] Gao X Tang M Tian S Li J. Liu W. A Ferroptosis-Related Gene Signature Predicts Overall Survival in Patients With Lung Adenocarcinoma. Future Oncol (2021) 17(12):1533–44. doi: 10.2217/fon-2020-1113 33432837

[18] Zhang A Yang J Ma C Li F Luo H. Development and Validation of a Robust Ferroptosis-Related Prognostic Signature in Lung Adenocarcinoma. Front Cell Dev Biol (2021) 9:616271. doi: 10.3389/fcell.2021.616271 34249899 PMC8264775

[19] Bersuker K Hendricks JM Li Z Magtanong L Ford B Tang PH. The CoQ Oxidoreductase FSP1 Acts Parallel to GPX4 to Inhibit Ferroptosis. Nature (2019) 575(7784):688–92. doi: 10.1038/s41586-019-1705-2 PMC6883167 31634900

[20] Doll S Freitas FP Shah R Aldrovandi M da Silva MC Ingold I. FSP1 is a Glutathione-Independent Ferroptosis Suppressor. Nature (2019) 575(7784):693–8. doi: 10.1038/s41586-019-1707-0 31634899

[21] Zhou N Bao J. FerrDb: A Manually Curated Resource for Regulators and Markers of Ferroptosis and Ferroptosis-Disease Associations. Database (Oxf) (2020) 2020:1–8. doi: 10.1093/database/baaa021 PMC7100629 32219413

[22] Tibshirani R. The Lasso Method for Variable Selection in the Cox Model. Stat Med (1997) 16(4):385–95. doi: 10.1002/(SICI)1097-0258(19970228)16:4<385::AID-SIM380>3.0.CO;2-3 9044528

[23] Newman AM Liu CL Green MR Gentles AJ Feng W Xu Y. Robust Enumeration of Cell Subsets From Tissue Expression Profiles. Nat Methods (2015) 12(5):453–7. doi: 10.1038/nmeth.3337 PMC4739640 25822800

[24] Yoshihara K Shahmoradgoli M Martínez E Vegesna R Kim H Torres-Garcia W. Inferring Tumour Purity and Stromal and Immune Cell Admixture From Expression Data. Nat Commun (2013) 4:2612. doi: 10.1038/ncomms3612 24113773 PMC3826632

[25] Charoentong P Finotello F Angelova M Mayer C Efremova M Rieder D. Pan-Cancer Immunogenomic Analyses Reveal Genotype-Immunophenotype Relationships and Predictors of Response to Checkpoint Blockade. Cell Rep (2017) 18(1):248–62. doi: 10.1016/j.celrep.2016.12.019 28052254

[26] Thorsson V Gibbs DL Brown SD Wolf D Bortone DS Ou Yang T-H. The Immune Landscape of Cancer. Immunity (2019) 51(2):411–2. 10.1016/j.immuni.2019.08.004 31433971

[27] Mayakonda A Lin D-C Assenov Y Plass C Koeffler HP. Maftools: Efficient and Comprehensive Analysis of Somatic Variants in Cancer. Genome Res (2018) 28(11):1747–56. doi: 10.1101/gr.239244.118 PMC6211645 30341162

[28] Kim JY Choi JK Jung H. Genome-Wide Methylation Patterns Predict Clinical Benefit of Immunotherapy in Lung Cancer. Clin Epigen (2020) 12(1):119. doi: 10.1186/s13148-020-00907-4 PMC7410160 32762727

[29] Hugo W Zaretsky JM Sun L Song C Moreno BH Hu-Lieskovan S. Genomic and Transcriptomic Features of Response to Anti-PD-1 Therapy in Metastatic Melanoma. Cell (2017) 168(3):542. doi: 10.1016/j.cell.2017.01.010 28129544

[30] Chen YP Wang YQ Lv JW Li YQ Chua MLK Le QT. Identification and Validation of Novel Microenvironment-Based Immune Molecular Subgroups of Head and Neck Squamous Cell Carcinoma: Implications for Immunotherapy. Ann Oncol (2019) 30(1):68–75. doi: 10.1093/annonc/mdy470 30407504

[31] Biswas SK Mantovani A. Macrophage Plasticity and Interaction With Lymphocyte Subsets: Cancer as a Paradigm. Nat Immunol (2010) 11(10):889–96. doi: 10.1038/ni.1937 20856220

[32] Hahn EE Gould MK. Lung Cancer Screening and Smoking Cessation: Never Too Early or Too Late. J Natl Cancer Inst (2018) 110(11):1157–8. doi: 10.1093/jnci/djy083 29788445

[33] Gong Z Jia Q Chen J Diao X Gao J Wang X. Impaired Cytolytic Activity and Loss of Clonal Neoantigens in Elderly Patients With Lung Adenocarcinoma. J Thorac Oncol (2019) 14(5):857–66. doi: 10.1016/j.jtho.2019.01.024 30768970

[34] Dogan S Shen R Ang DC Johnson ML D'Angelo SP Paik PK. Molecular Epidemiology of EGFR and KRAS Mutations in 3,026 Lung Adenocarcinomas: Higher Susceptibility of Women to Smoking-Related KRAS-Mutant Cancers. Clin Cancer Res An Off J Am Assoc Cancer Res (2012) 18(22):6169–77. doi: 10.1158/1078-0432.CCR-11-3265 PMC3500422 23014527

[35] Kadara H Choi M Zhang J Parra ER Rodriguez-Canales J Gaffney SG. Whole-Exome Sequencing and Immune Profiling of Early-Stage Lung Adenocarcinoma With Fully Annotated Clinical Follow-Up. Ann Oncol (2018) 29(4):1072. doi: 10.1093/annonc/mdx062 29688333 PMC6887935

[36] Chen H Yang M Wang Q Song F Li X Chen K. The New Identified Biomarkers Determine Sensitivity to Immune Check-Point Blockade Therapies in Melanoma. Oncoimmunology (2019) 8(8):1608132. doi: 10.1080/2162402X.2019.1608132 31413919 PMC6682357

[37] Pinsky PF. Cost-Effectiveness of CT Screening in the National Lung Screening Trial. N Engl J Med (2015) 372(4):387. doi: 10.1056/NEJMc1414726 25607439

[38] Chen Y Li Z-Y Zhou G-Q Sun Y. An Immune-Related Gene Prognostic Index for Head and Neck Squamous Cell Carcinoma. Clin Cancer Res An Off J Am Assoc Cancer Res (2021) 27(1):330–41. doi: 10.1158/1078-0432.CCR-20-2166 33097495

[39] Galon J Bruni D. Approaches to Treat Immune Hot, Altered and Cold Tumours With Combination Immunotherapies. Nat Rev Drug Discovery (2019) 18(3):197–218. doi: 10.1038/s41573-018-0007-y 30610226

[40] Topalian SL Taube JM Anders RA Pardoll DM. Mechanism-Driven Biomarkers to Guide Immune Checkpoint Blockade in Cancer Therapy. Nat Rev Cancer (2016) 16(5):275–87. doi: 10.1038/nrc.2016.36 PMC5381938 27079802

[41] Zeng D Ye Z Wu J Zhou R Fan X Wang G. Macrophage Correlates With Immunophenotype and Predicts Anti-PD-L1 Response of Urothelial Cancer. Theranostics (2020) 10(15):7002–14. doi: 10.7150/thno.46176 PMC7295060 32550918

[42] Fridman WH Zitvogel L Sautès-Fridman C Kroemer G. The Immune Contexture in Cancer Prognosis and Treatment. Nat Rev Clin Oncol (2017) 14(12):717–34. doi: 10.1038/nrclinonc.2017.101 28741618

[43] Ruffell B Coussens LM. Macrophages and Therapeutic Resistance in Cancer. Cancer Cell (2015) 27(4):462–72. doi: 10.1016/j.ccell.2015.02.015 PMC4400235 25858805

[44] Hansen AR Siu LL. PD-L1 Testing in Cancer: Challenges in Companion Diagnostic Development. JAMA Oncol (2016) 2(1):15–6. doi: 10.1001/jamaoncol.2015.4685 26562503

[45] Yarchoan M Hopkins A Jaffee EM. Tumor Mutational Burden and Response Rate to PD-1 Inhibition. N Engl J Med (2017) 377(25):2500–1. doi: 10.1056/NEJMc1713444 PMC6549688 29262275

[46] Jiang P Gu S Pan D Fu J Sahu A Hu X. Signatures of T Cell Dysfunction and Exclusion Predict Cancer Immunotherapy Response. Nat Med (2018) 24(10):1550–8. doi: 10.1038/s41591-018-0136-1 PMC6487502 30127393

[47] Liao W Overman MJ Boutin AT Shang X Zhao D Dey P. KRAS-IRF2 Axis Drives Immune Suppression and Immune Therapy Resistance in Colorectal Cancer. Cancer Cell (2019) 35(4):559–72.e7. doi: 10.1016/j.ccell.2019.02.008 PMC6467776 30905761

[48] Shi L Liu Y Li M Luo Z. Emerging Roles of Ferroptosis in the Tumor Immune Landscape: From Danger Signals to Anti-Tumor Immunity. FEBS J (2021). doi: 10.1111/febs.16034 34042258

[49] Ma X Xiao L Liu L Ye L Su P Bi E. CD36-Mediated Ferroptosis Dampens Intratumoral CD8 T Cell Effector Function and Impairs Their Antitumor Ability. Cell Metab (2021) 33(5):1001–12.e5. doi: 10.1016/j.cmet.2021.02.015 PMC8102368 33691090

