# Supplementary figures and images for: Clinical Significance and Immune Landscape of Recurrence-Associated Ferroptosis Signature in Early-Stage Lung Adenocarcinoma
Source: Front Oncol. 2022 Jan 27;12:794293. doi: 10.3389/fonc.2022.794293 (PMC8828635; doi:10.3389/fonc.2022.794293)

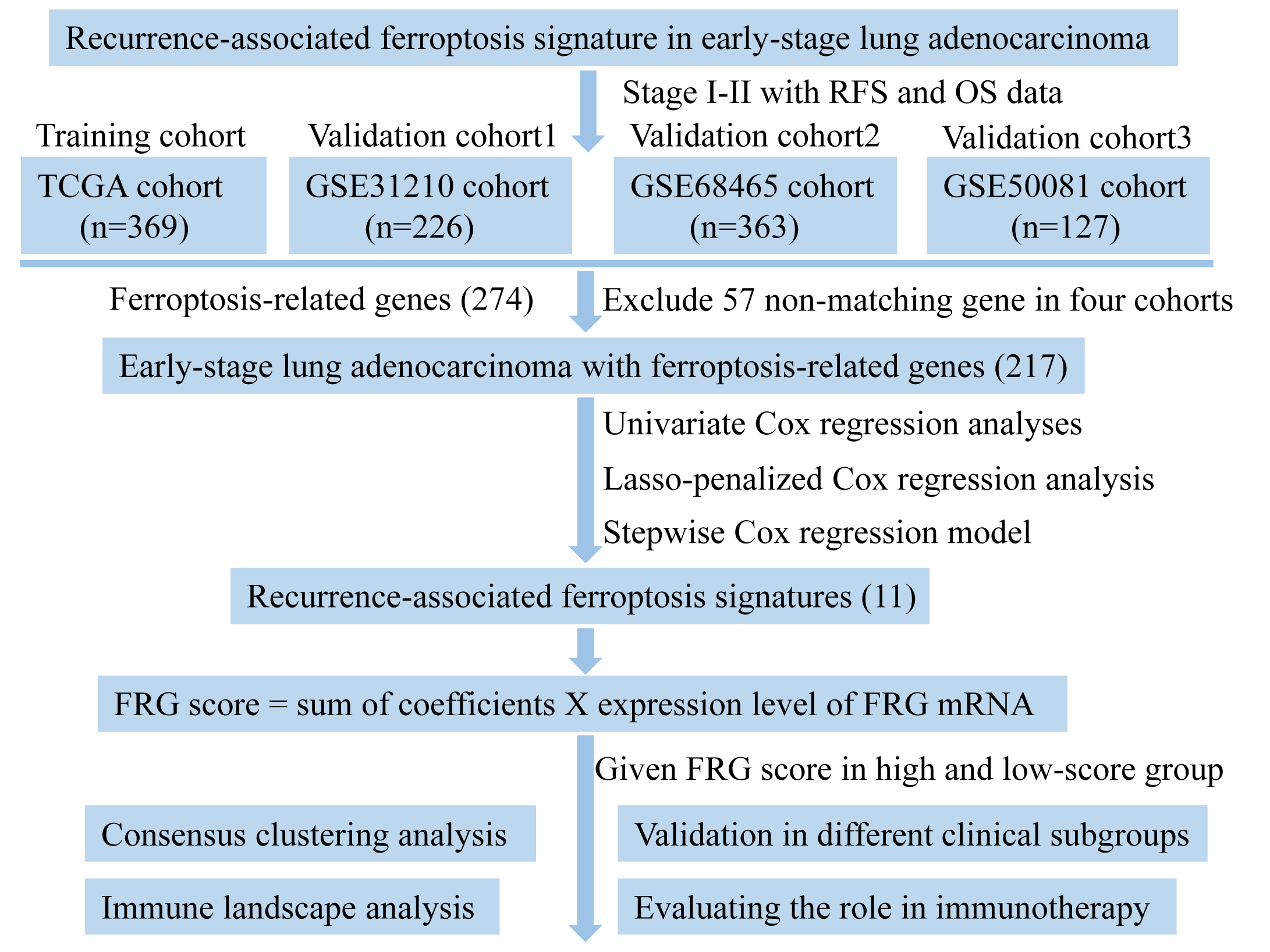

Supplement: Supplementary Figure 1 — A pipeline for generation and validation of FRG signatures. [file Image_1.tif]

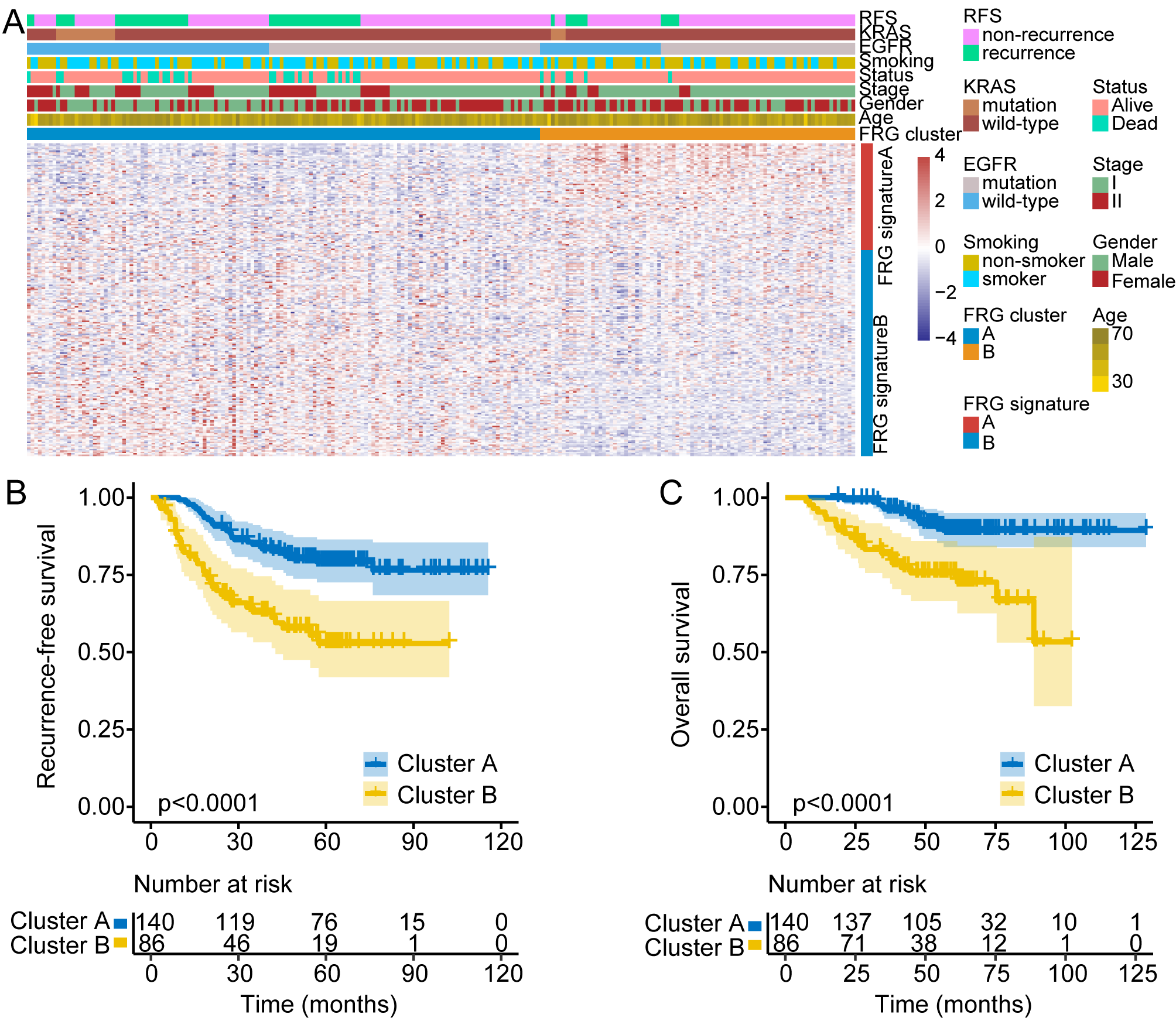

Supplement: Supplementary Figure 2 — Validation of FRGs-based subtypes in GSE31210 cohort. (A) Unsupervised clustering of FRGs. (B, C) Kaplan-Meier curves of recurrence-free survival (B) and overall survival (C) for early-stage LUAD patients in two FRG-based clusters. [file Image_2.tif]

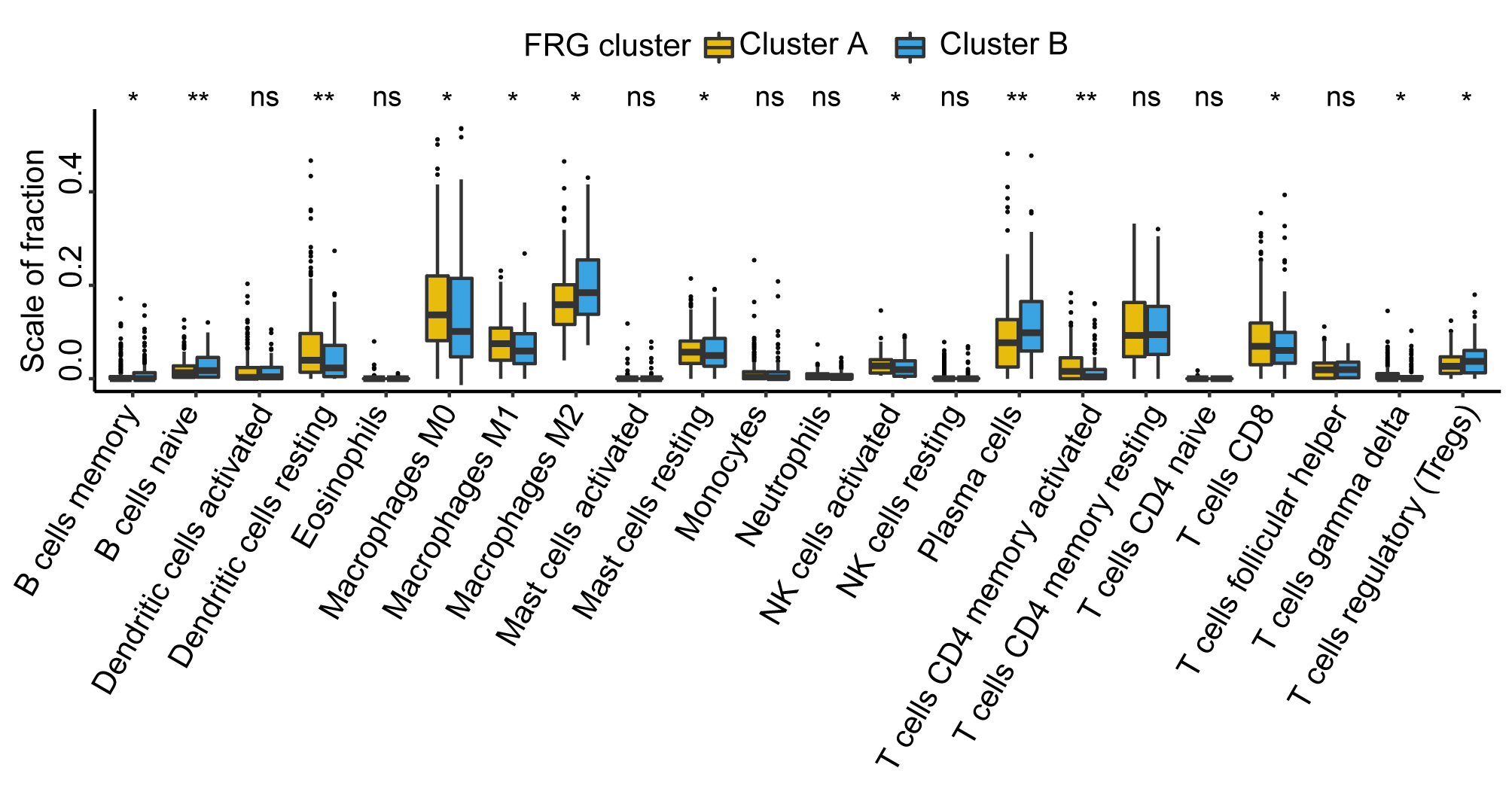

Supplement: Supplementary Figure 3 — Levels of 22 infiltrating immune cells in two FRG-based clusters determined by CIBERSORT algorithm. ns, no significance; *p < 0.05; **p < 0.01. [file Image_3.tif]

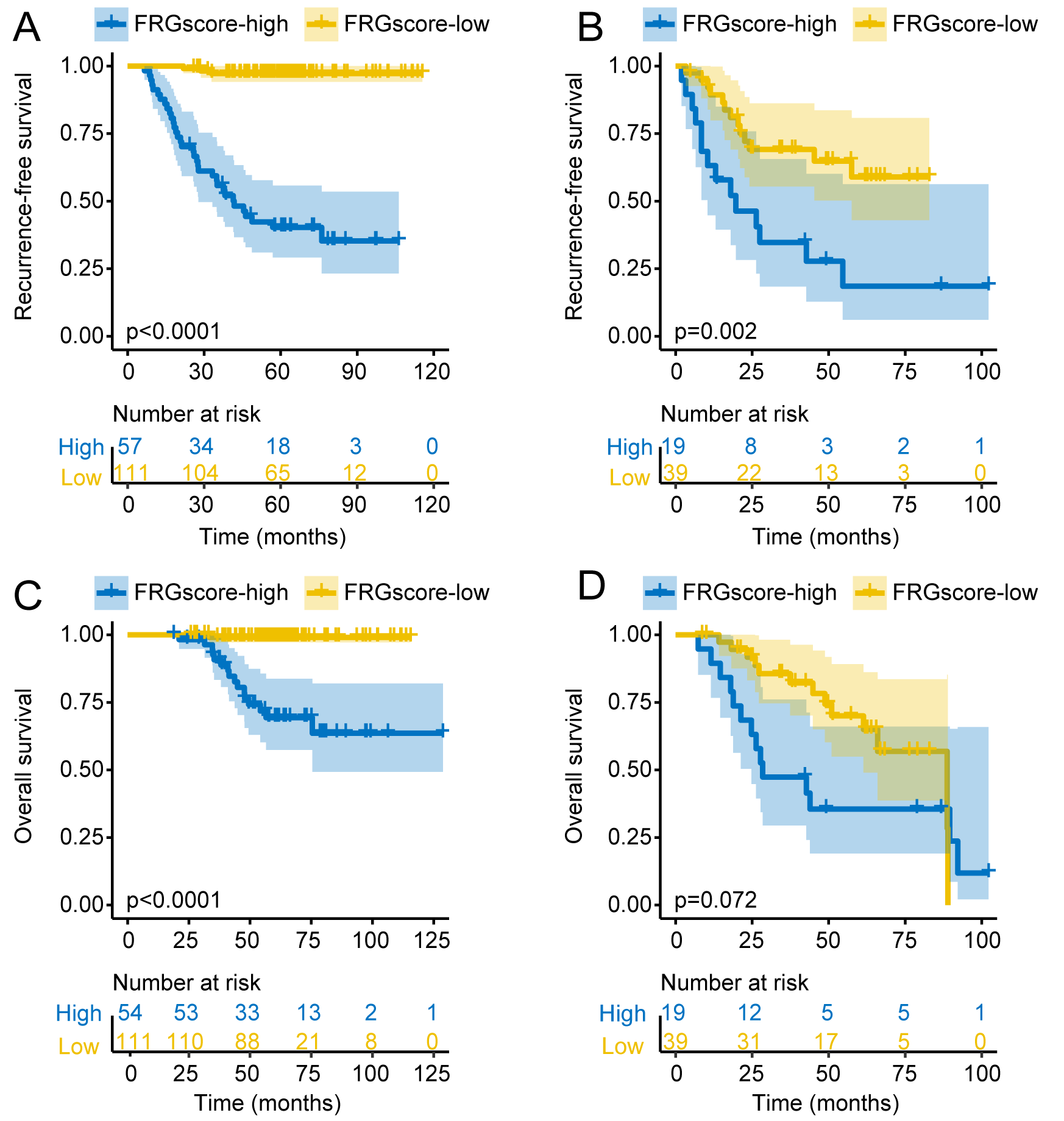

Supplement: Supplementary Figure 4 — Survival curves of FRGscore in GSE31210 cohort. (A-B) Kaplan-Meier recurrence-free survival curves in stage I (A) and stage II (B) LUAD patients based on FRGscore. (C, D) Kaplan-Meier overall survival curves in stage I (C) and stage II (D) LUAD patients based on FRGscore. [file Image_4.tif]

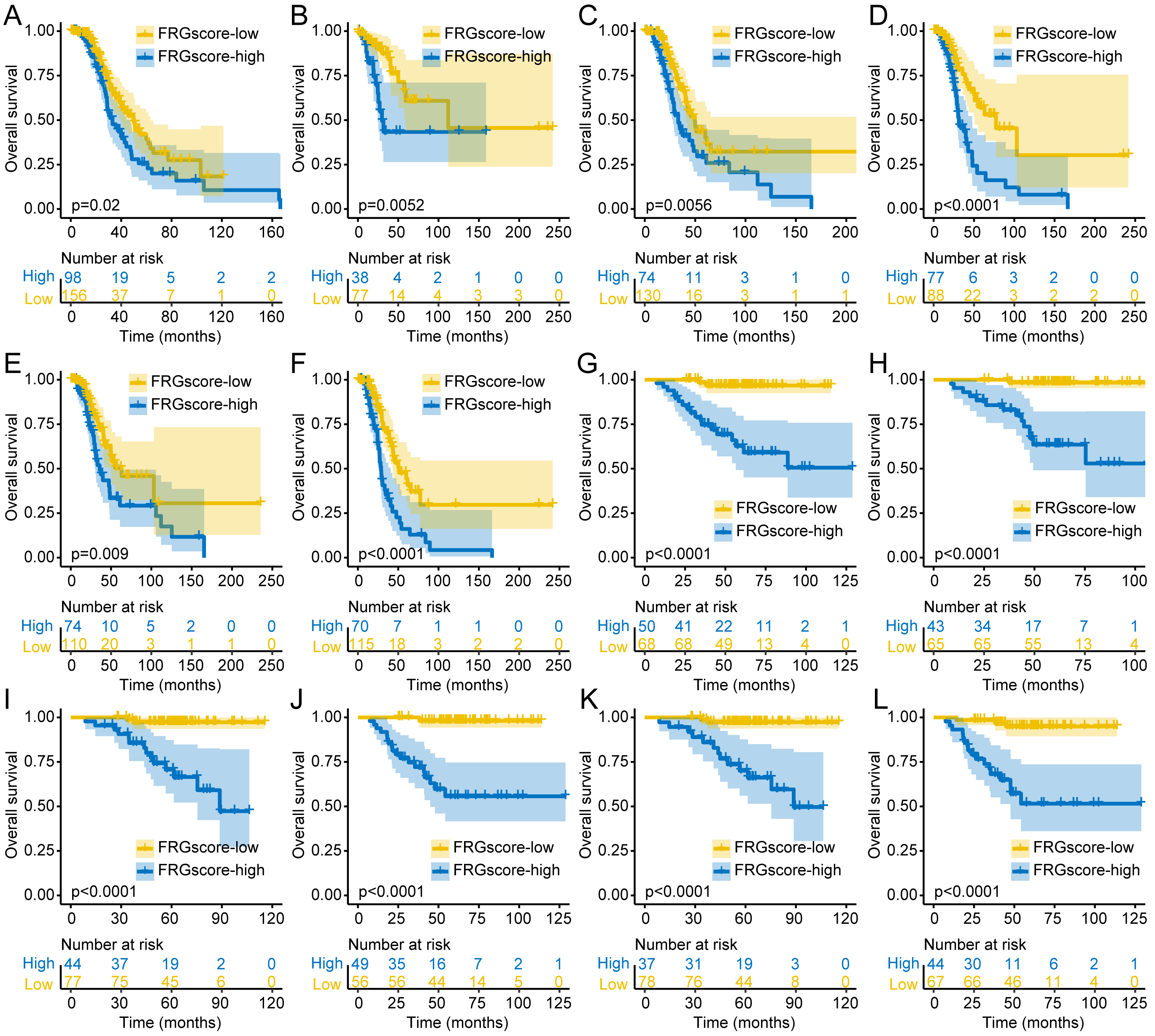

Supplement: Supplementary Figure 5 — Survival analysis of early-stage LUAD patients stratified by age, gender and smoking history. (A–F) Kaplan-Meier curves of overall survival in older (A), younger (B), female (C), male (D), non-smokers (E) and smokers (F) patients based on FRGscore in TCGA cohort. (G-L) Kaplan-Meier curves of overall survival in older (G), younger (H), female (I), male (J), non-smokers (K) and smokers (L) patients based on FRGscore in GSE31210 cohort. [file Image_5.tif]

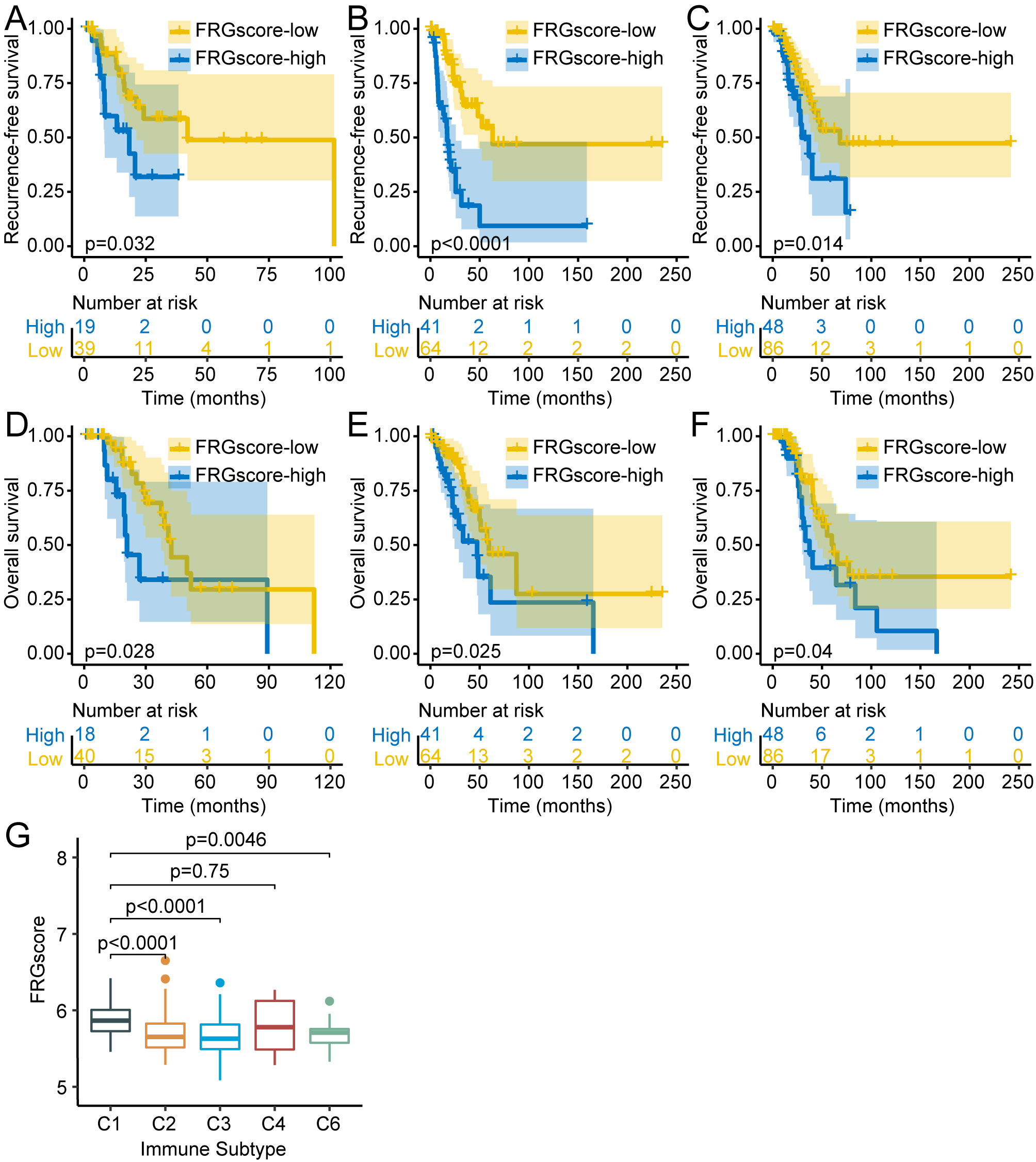

Supplement: Supplementary Figure 6 — Survival analysis of early-stage LUAD patients stratified by immune subtypes in TCGA cohort. (A–C) Kaplan-Meier curves of recurrence-free survival in C1 (A), C2 (B) and C3 (C) immune subtypes based on FRGscore. (D–F) Kaplan-Meier curves of overall survival in C1 (D), C2 (E) and C3 (F) immune subtypes based on FRGscore. (G) Distribution of FRGscore in different immune subtypes. [file Image_6.tif]

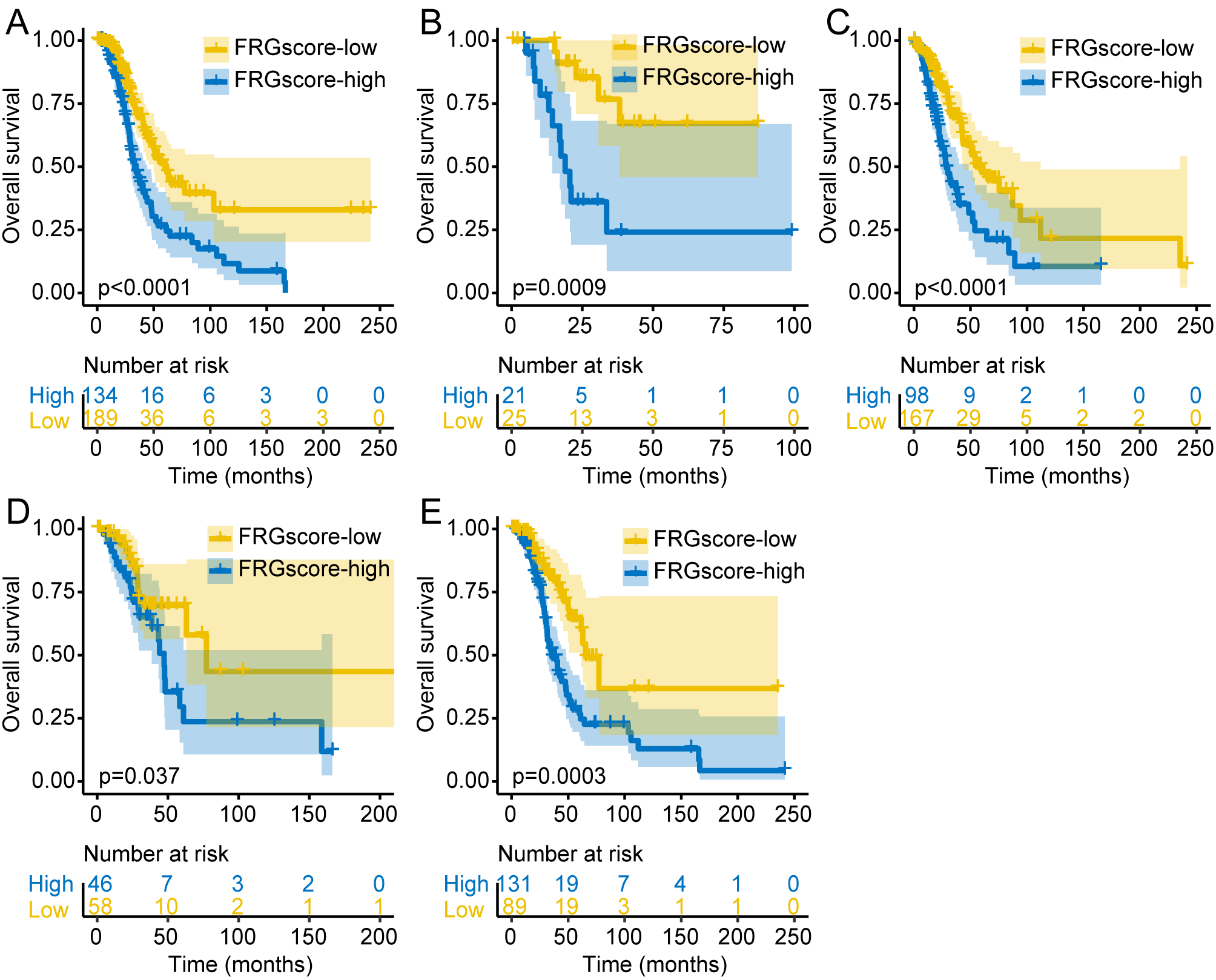

Supplement: Supplementary Figure 7 — Survival analysis of early-stage LUAD patients harboring EGFR or KRAS mutation in TCGA cohort. Kaplan-Meier curves of overall survival in EGFR-WT (A), EGFR-Mut (B), KRAS-WT (C), KRAS-Mut (D) and EGFR/KRAS-WT (E) patients based on FRGscore. [file Image_7.tif]

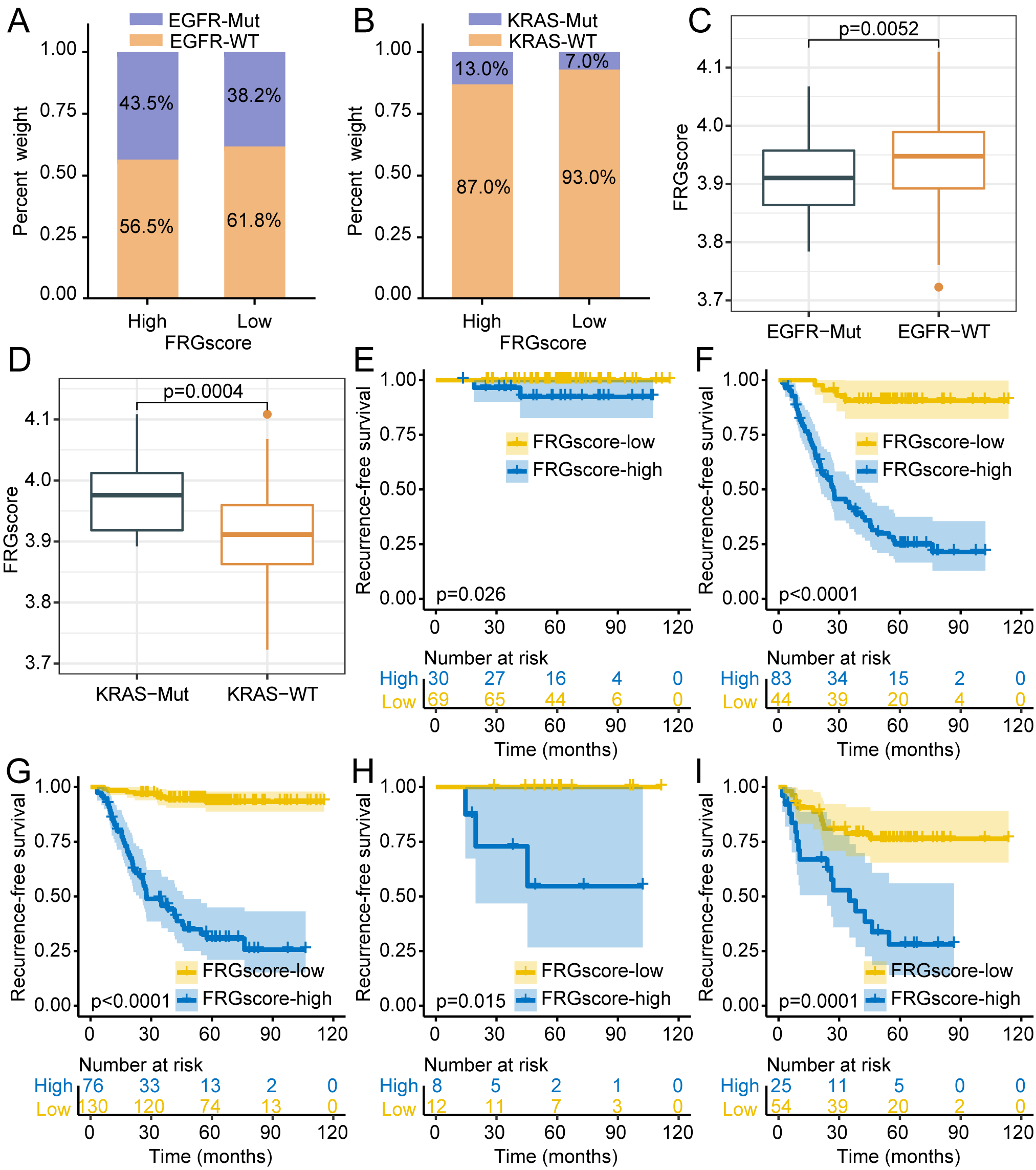

Supplement: Supplementary Figure 8 — Validation of FRGscore in different molecular subgroups in GSE31210 cohort. (A, B) Proportion of patients harboring EGFR (A) and KRAS (B) mutations in FRGscore-high or FRGscore-low subgroups. (C–D) Distribution of FRGscore in different mutation status of EGFR (C) and KRAS (D). (E–I) Kaplan-Meier curves of recurrence-free survival in EGFR-WT (E), EGFR-Mut (F), KRAS-WT (G), KRAS-Mut (H) and EGFR/KRAS-WT (I) patients based on FRGscore. [file Image_8.tif]

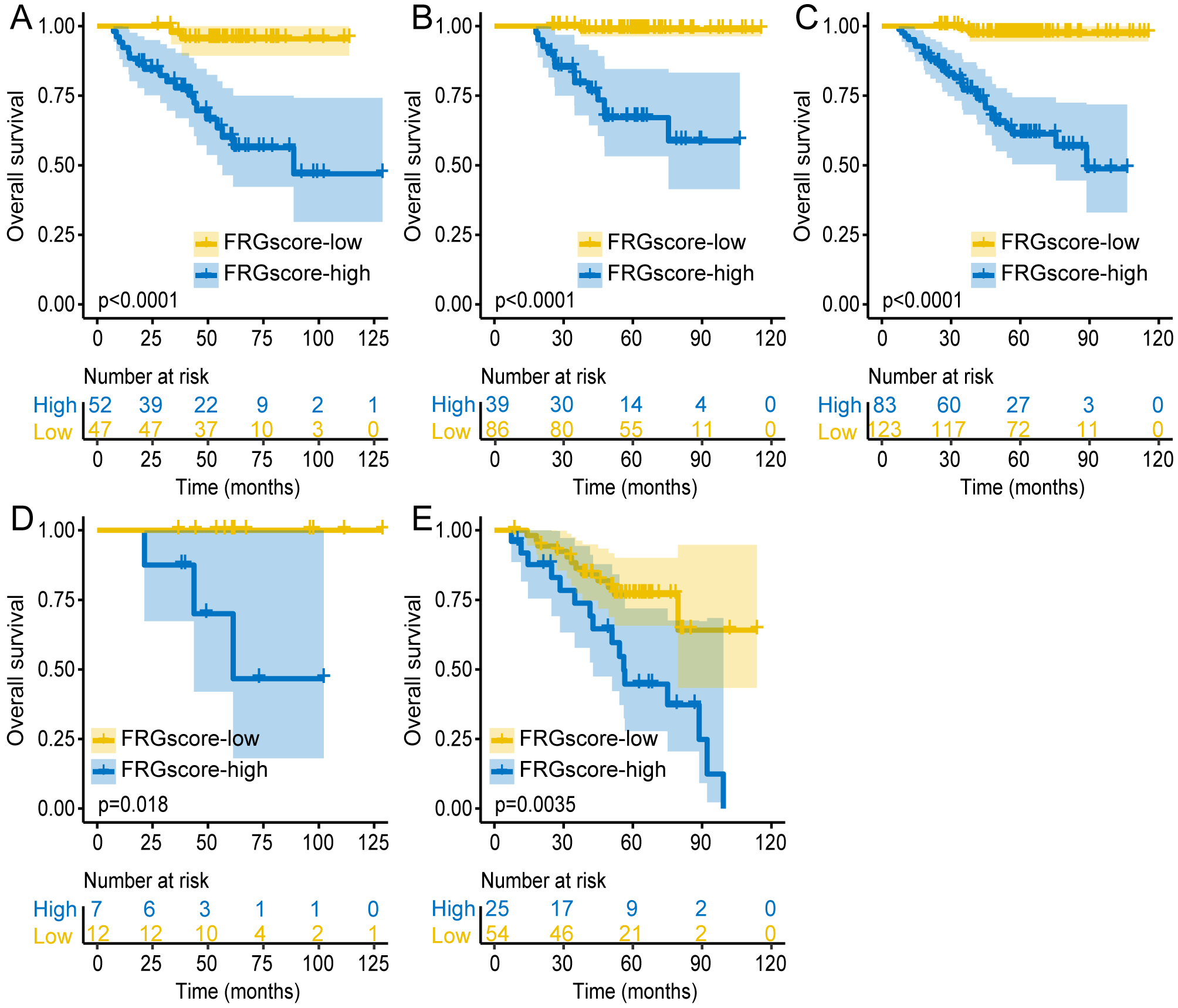

Supplement: Supplementary Figure 9 — Survival analysis of early-stage LUAD patients harboring EGFR or KRAS mutation in GSE31210 cohort. Kaplan-Meier curves of overall survival in EGFR-WT (A), EGFR-Mut (B), KRAS-WT (C), KRAS-Mut (D) and EGFR/KRAS-WT (E) patients based on FRGscore. [file Image_9.tif]

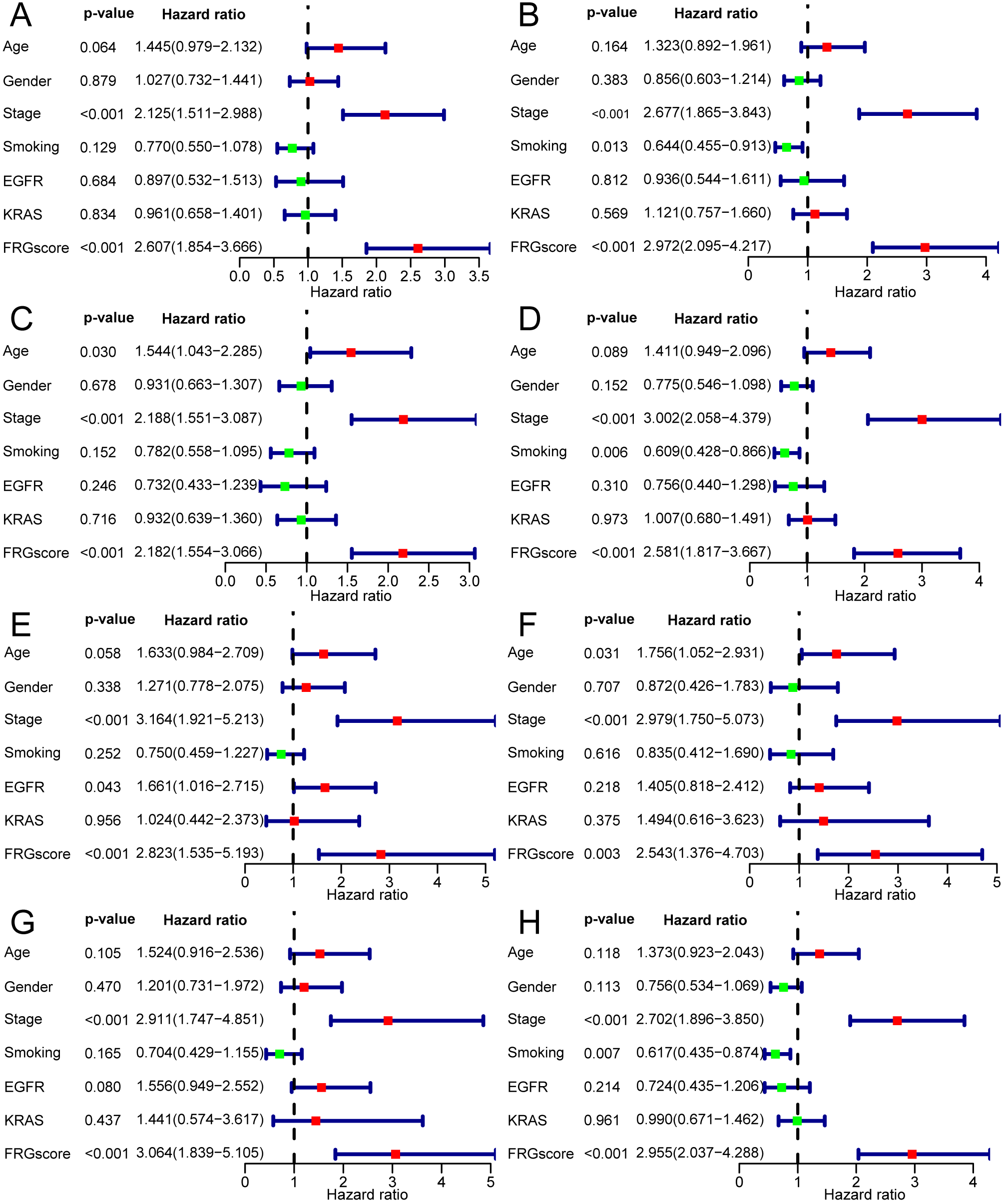

Supplement: Supplementary Figure 10 — Independent prognostic value of FRGscore. (A, B) Univariate (A) and multivariate (B) Cox analyses of recurrence-free survival based on FRGscore in TCGA cohort. (C–D) Univariate (C) and multivariate (D) Cox analyses of overall survival based on FRGscore in TCGA cohort. (E-F) Univariate (E) and multivariate (F) Cox analyses of recurrence-free survival based on FRGscore in GSE31210 cohort. (G, H) Univariate (G) and multivariate (H) Cox analyses of overall survival based on FRGscore in GSE31210 cohort. [file Image_10.tif]

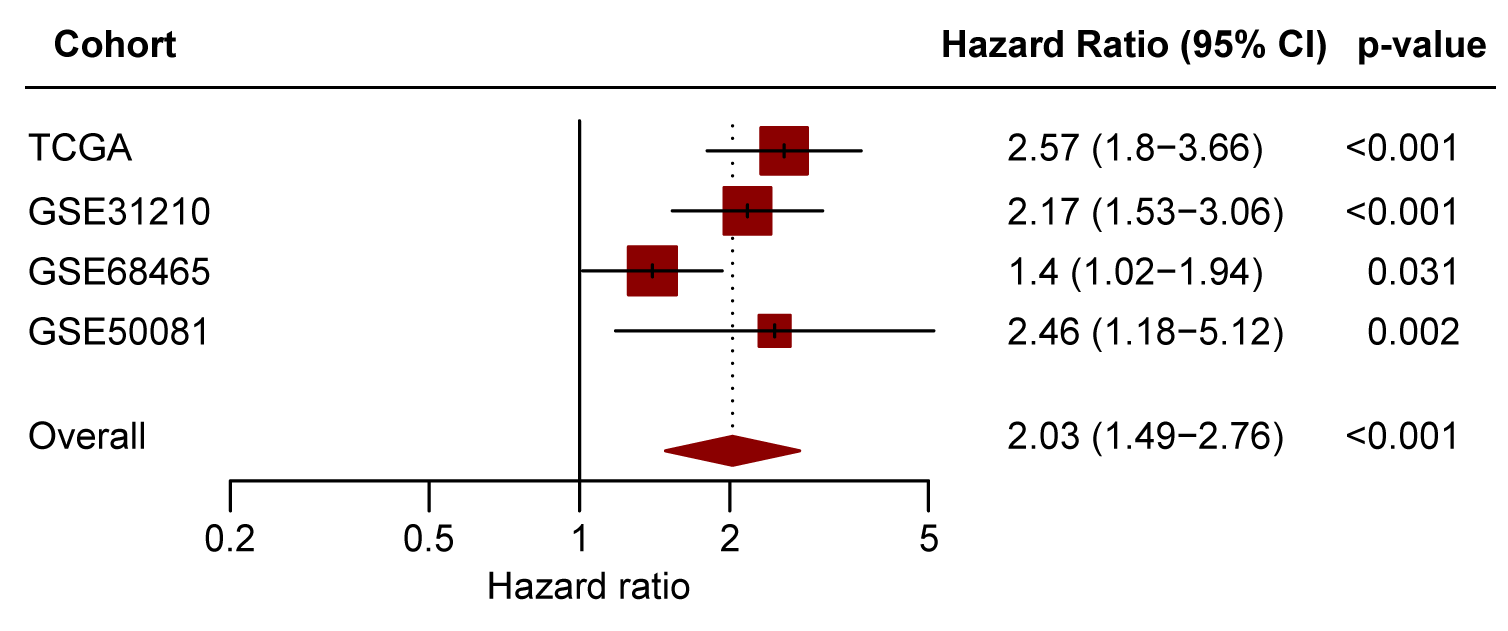

Supplement: Supplementary Figure 11 — Meta-analysis of prognostic values of FRG signatures in early-stage LUAD patients across the four cohorts. P-values of TCGA (n=369), GSE31210 (n=226), GSE68465 (n=363) and GSE50081 (n=127) were calculated by Kaplan-Meier curves. P-value of overall (n=1085) patients was calculated by meta-analysis. [file Image_11.tif]

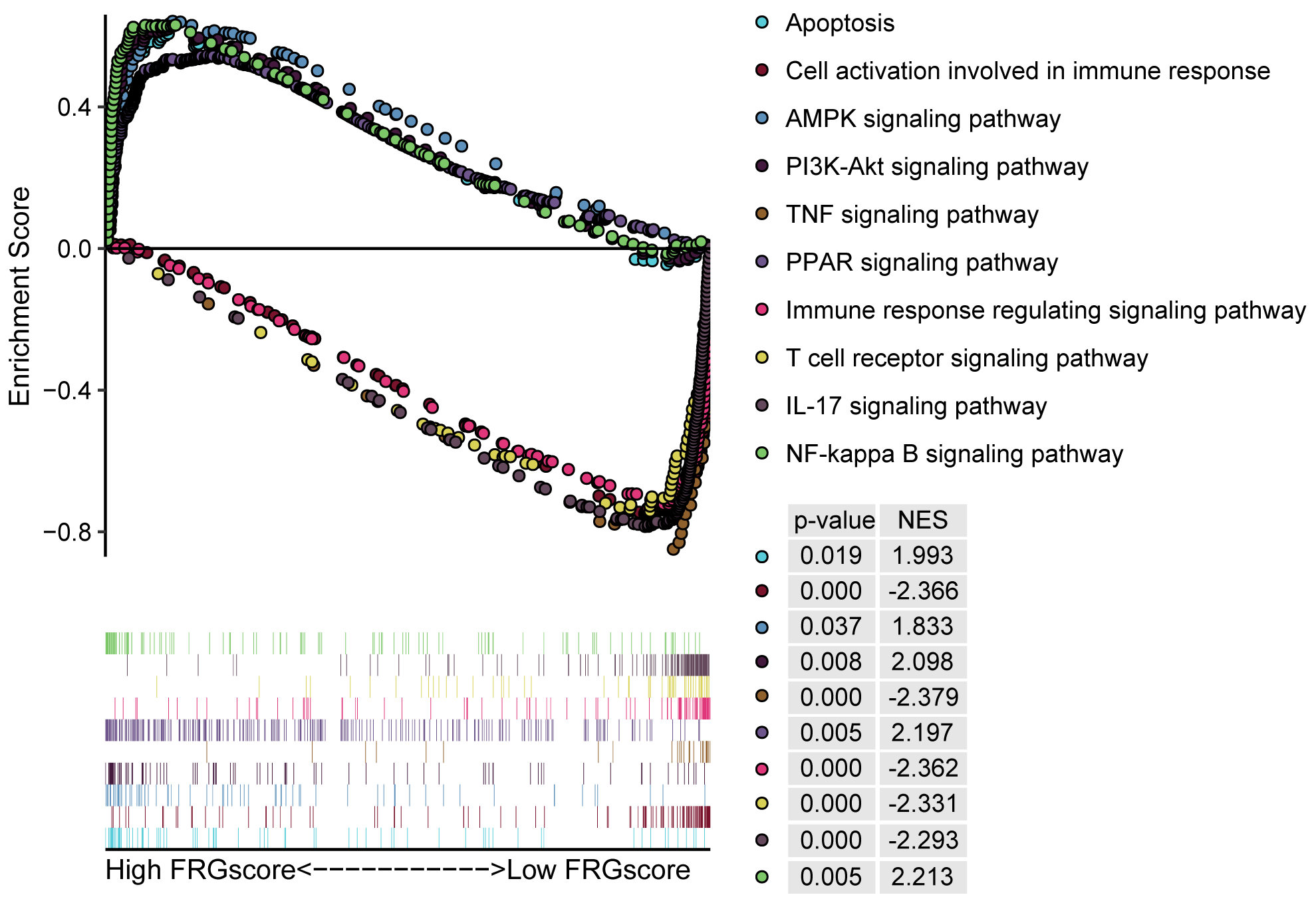

Supplement: Supplementary Figure 12 — Biological pathways associated with FRGscore-high and FRGscore-low subgroups. [file Image_12.tif]

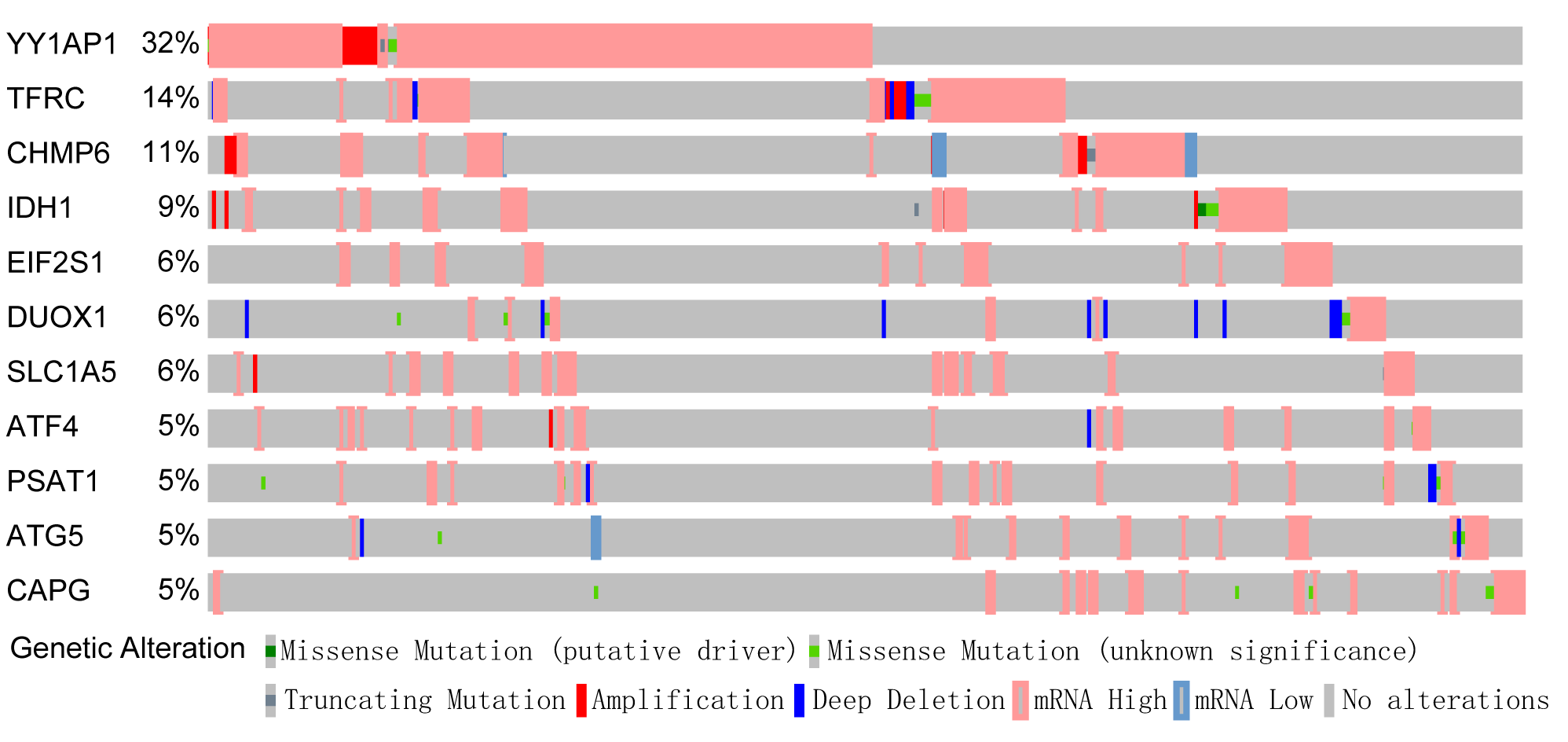

Supplement: Supplementary Figure 13 — Genetic alteration of the identified 11 FRG signatures in TCGA-LUAD cohort. [file Image_13.tif]
